# High performance polymerized small molecule acceptor by synergistic optimization on π-bridge linker and side chain

**DOI:** 10.1038/s41467-022-32964-z

**Published:** 2022-09-07

**Authors:** Guangpei Sun, Xin Jiang, Xiaojun Li, Lei Meng, Jinyuan Zhang, Shucheng Qin, Xiaolei Kong, Jing Li, Jingming Xin, Wei Ma, Yongfang Li

**Affiliations:** 1 Beijing National Laboratory for Molecular Sciences, CAS Key Laboratory of Organic Solids, Institute of Chemistry, Chinese Academy of Sciences, Beijing, 100190 China; 2 School of Chemical Science, University of Chinese Academy of Sciences, Beijing, 100049 China; 3 Key Laboratory of Photochemical Conversion and Optoelectronic Materials, Technical Institute of Physics and Chemistry, Chinese Academy of Sciences, Beijing, 100190 China; 4 State Key Laboratory for Mechanical Behavior of Materials, Xi’an Jiaotong University, Xi’an, 710049 P. R. China; 5 Laboratory of Advanced Optoelectronic Materials, Suzhou Key Laboratory of Novel Semiconductor Materials and Devices, College of Chemistry, Chemical Engineering and Materials Science, Soochow University, Suzhou, Jiangsu 215123 China

**Keywords:** Solar cells, Conjugated polymers, Solar cells

## Abstract

The polymerized small-molecule acceptors have attracted great attention for application as polymer acceptor in all-polymer solar cells recently. The modification of small molecule acceptor building block and the π-bridge linker is an effective strategy to improve the photovoltaic performance of the polymer acceptors. In this work, we synthesized a new polymer acceptor PG-IT2F which is a modification of the representative polymer acceptor PY-IT by replacing its upper linear alkyl side chains on the small molecule building block with branched alkyl chains and attaching difluorene substituents on its thiophene π-bridge linker. Through this synergistic optimization, PG-IT2F possesses more suitable phase separation, increased charge transportation, better exciton dissociation, lower bimolecular recombination, and longer charge transfer state lifetime than PY-IT in their polymer solar cells with PM6 as polymer donor. Therefore, the devices based on PM6:PG-IT2F demonstrated a high power conversion efficiency of 17.24%, which is one of the highest efficiency reported for the binary all polymer solar cells to date. This work indicates that the synergistic regulation of small molecule acceptor building block and π-bridge linker plays a key role in designing and developing highly efficient polymer acceptors.

## Abstract (web summary)

The modification of small molecule acceptor building block and π−bridge linker is effective to improve photovoltaic performance. Here, the authors replace linear with branched alkyl chains and introduce difluorene-substituted linker to realise all-polymer solar cells with efficiency of 17.24%.

## Introduction

Polymer solar cells (PSCs) have attracted extensive attention in recent years, on account of their advantages of simple device structure with solution processing, lightweight, translucent properties, and mechanical flexibility^1–4^. Recently, the PSCs based on wide bandgap polymer donor and narrow bandgap small-molecule acceptor (SMA) have achieved power conversion efficiency (PCE) over 18%^5–7^. In comparison with the SMAs-based PSCs, all-polymer solar cells (all-PSCs) with *n*-type conjugated polymer as acceptor have their unique merits of good morphological stability, high light-irradiation stability and greater mechanical stress strength, which make them more suitable for the application of flexible devices^8–11^. However, the PCE of all-PSCs lags behind that of the SMAs-based PSCs due to the lack of high performance polymer acceptors in early research^12,13^

In 2017, a strategy known as polymerized small molecule acceptor (PSMA) was proposed by Li and Zhang et al.^14^, which is composed of a narrow bandgap SMA as main building block copolymerized with a π-bridge linking unit (or called as linker). This strategy conferred the polymer acceptors to the advantages of SMAs with broad and strong absorption and suitable electronic energy levels, in combination of the advantages of the polymers with good mechanical flexibility and high thermal and opto-stability^15^. Therefore, the studies on PSMAs have attracted great attention in recent years. Especially, after the report of the A-DA’D-A structured SMA Y6 in 2019^16^, the PCE of the all-PSCs based on the PSMAs with Y6 or its derivatives as SMA building block (like PY-IT) is boosted to over 15%^17–19^.

The optimization of narrow bandgap SMA building block has drawn great attention to improve the photovoltaic performance of PSMAs^20,21^, while less attention has been paid to its π-bridge linking units^22–24^. Currently, most of the PSMAs use unsubstituted thiophene, selenophene^25^ or BDT^26,27^ as π-bridge linker. At present, the efficiency of the all-PSCs still lags the SMAs-based devices, which is generally due to the lower FF caused by difficult regulation of morphology and insufficient electron mobilities. As a matter of fact, in addition to improving the morphology of PSMAs, π-bridge linking units influence the exciton dissociation, charge extraction, and charge transport as well^28,29^. Thus, modification of the π-bridge linker could be an important strategy to improve the photovoltaic performance of the PSMAs.

It has been shown that the incorporation of electron-withdrawing F substituents on polymer backbone can afford decent *n*-type properties of the derived polymers, since the F atom can effectively increase the electron affinity of conjugated polymer to facilitate electron transport and stabilize the formed radical anions on the polymer backbone^30,31^. Meanwhile, the intra- and inter-molecular hydrogen bonding F∙∙∙H can also promote the molecular organization to enhance their charge carrier mobility^32^. Therefore, introducing F atom into π-bridge of PMSAs may be one of the methods to improve the electron transport property of the polymer acceptors. Nevertheless, fluorination is not omnipotent, it may also bring some problems such as affecting the conformation, miscibility, and crystallinity of the polymers^33,34^. Therefore, while introducing F atom into the π-bridge linker of the PSMAs, it may be necessary to simultaneously regulate the molecular structure of the SMA backbone in order to achieve the photovoltaic property optimization of the PSMAs.

**Fig. 1:**
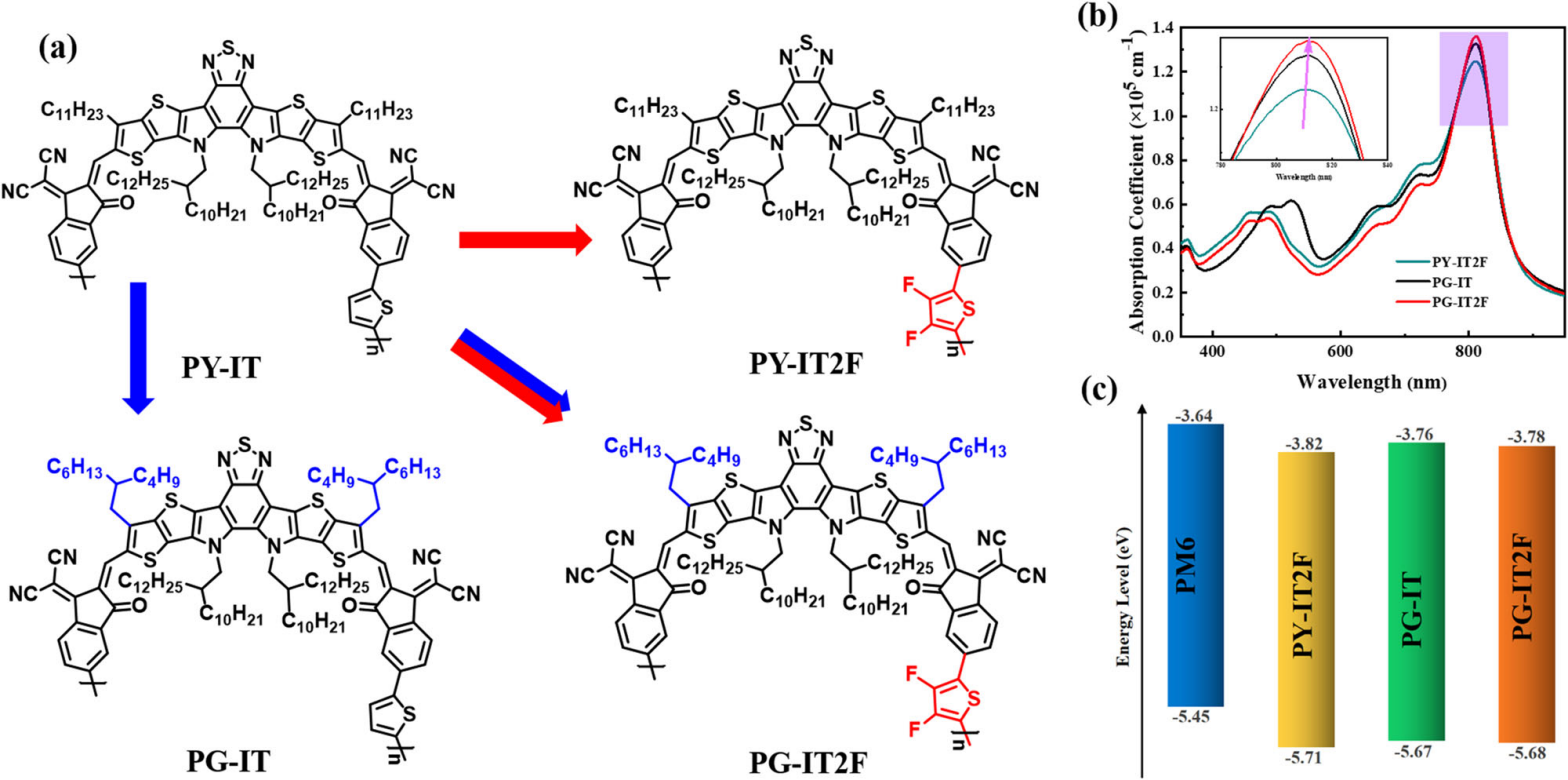
Molecular structures and physicochemical properties of PSMAs. **a** Molecular structures (blue color alkyl chains are the branched upper side chains, and red color difluorothiophene is the modified linking unit) of PY-IT, PY-IT2F, PG-IT, and PG-IT2F. **b** Absorption spectra of the PSMAs films. **c** Energy level diagram of the PSMAs and PM6 donor. Source data are provided as a Source Data file.

In 2020, Yang et al.^17^ synthesized a PSMA PY-IT with a regular (inner) connection between the SMA molecular backbone and the thiophene linker in the polymer, which shows a red-shifted absorption and higher PCE of 15.05% for the all-PSCs with PM6 as donor. Here, we studied the synergistic effect of fluorinated thiophene π-bridge linker and branched upper alkyl side chains of the SMA backbone on the photovoltaic properties of PY-IT, by synthesizing a new PSMA PG-IT2F (see the molecular structure in Fig. 1a). First, we introduced 3,4-difluorothiophene π-bridge linking unit into PY-IT and synthesized PY-IT2F (see Fig. 1a), to enhance the electron mobility of the PMSA for improving the FF of the corresponding all-PSCs. However, although the electron mobility of PY-IT2F is improved, the *J*_sc_ and FF of the all-PSC based on PM6:PY-IT2F are relatively low because there is serious charge carrier recombination in the active layer due to the excessive miscibility of PY-IT2F with PM6. In considering that the branched alkyl upper side chains on the fused ring of Y6 derivatives significantly influence the molecular packing of the SMA^35^ and may decrease the miscibility of the SMA with PM6^36^, we introduced the branched side chains on the SMA backbone to adjust the morphology of active layer, and synthesized the PSMAs PG-IT and PG-IT2F (see Fig. 1a). We found that PG-IT2F possesses suitable miscibility with PM6, higher electron mobility, and lower charge carrier recombination. The binary all-PSCs based on PM6:PG-IT2F demonstrated a high PCE of 17.24% with an open circuit voltage (*V*_oc_) of 0.95 V, a short circuit current density (*J*_sc_) of 24.03 mA cm^−2^, and a higher fill factor (FF) of 75.46%, which is one of the highest reported PCE among the binary all-PSCs.

## Results

### Synthesis and physicochemical properties of the PSMAs

Figure 1a shows the molecular structures of the PSMAs of PY-IT, PY-IT2F, PG-IT, and PG-IT2F, their synthetic routes are shown in Supplementary Figs. 1–5. PY-IT was synthesized according to the literature method^17^. PY-IT2F, PG-IT, and PG-IT2F were synthesized by Stille cross-coupling polymerization of the brominated monomer 1 or monomer 6 (see NMR data in Supplementary Figs. 6–11) and the 2,5-bis (trimethylstannyl) bifluorothiophene or 2,5-bis (trimethylstannyl) thiophene linking units, and their detailed synthetic processes were described in Supplementary Methods. The number average molecular weights (*M*_n_) of PY-IT2F, PG-IT, and PG-IT2F were measured to be 26.3 kDa, 11.9 kDa, and 17.1 kDa with a polydispersity index (PDIs) of 3.2, 2.4, and 2.7, respectively, by the high-temperature gel-permeation chromatography (GPC), as shown in Supplementary Fig. 12. Thermogravimetric analysis was performed to measure the thermal stabilities of these acceptors, and all the PSMAs exhibit decomposition temperatures of ~340 °C with 5% weight loss under nitrogen atmosphere (Supplementary Fig. 13), which indicates that thermal stability of these materials is high enough for the application in all-PSCs.

The normalized UV-vis absorption spectra of PY-IT2F, PG-IT, and PG-IT2F in solution and blend films are shown in Supplementary Fig. 14. The detailed data of the optical properties were summarized in Table 1. Figure 1b shows the absorption spectra of the PSMAs films. The main absorption peaks of PY-IT2F, PG-IT, and PG-IT2F films are at 811, 803, and 811 nm, respectively. Apparently, the introduction of difluorothiophene π-bridge linker makes the absorption peaks of PY-IT2F and PG-IT2F films slightly red-shifted than that of PY-IT and PG-IT films. In addition, as shown in Fig. 1b, the peak absorption coefficients of the PSMA films increase from PY-IT2F to PG-IT to PG-IT2F, which indicates that the introduction of the fluorinated π-bridge linker and branched upper alkyl side chains are all helpful to enhance the light absorption ability of the PSMAs.

**Table 1:** Physicochemical properties of PY-IT2F, PG-IT, and PG-IT2F

PSMAs	*λ*_max_ solution (nm)	*λ*_max_ film (nm)	*λ*_onset_ film (nm)	*ε*_*max*_ film (10^5^ cm^−1^)		${E}_{g}^{{opt}}$ (eV)^a^	*E*_HOMO_ (eV)	*E*_LUMO_ (eV)
PY-IT2F	784	811	895		1.25	1.38	−5.71	−3.82
PG-IT	782	803	888		1.33	1.40	−5.67	−3.76
PG-IT2F	788	811	890		1.36	1.39	−5.68	−3.78

^a^Calculated from the absorption edge of the polymer films: ${E}_{g}^{{opt}}$= 1240/*λ*_onset_.

The highest occupied molecular orbital (HOMO) and the lowest unoccupied molecular orbital (LUMO) energy levels (*E*_HOMO/LUMO_) of the PSMAs were measured by cyclic voltammetry with Ag/AgCl as reference electrode, and were calculated according to the equations of

1 $${E}_{{{{{{\rm{HOMO}}}}}}/{{{{{\rm{LUMO}}}}}}}=-e({\varphi}_{{{{{{\rm{ox}}}}}}/{{{{{\rm{red}}}}}}}+4.38)(eV)$$

where *φ*_ox/red_ values are the onset oxidation/reduction potentials (vs. Ag/AgCl) obtained from the cyclic voltammograms (CVs) as shown in Supplementary Fig. 15. Compared with PY-IT^17^, PY-IT2F has down-shifted HOMO and LUMO energy levels (see Table 1), which is expected because of the fluorination^31^. The HOMO and LUMO energy levels of PG-IT are −5.67 and −3.76 eV, respectively, which are similar with that of PY-IT. This result indicates that the branched side chain substitution has little effect on the energy level of PSMAs. For the PG-IT2F, the values of *E*_HOMO_/*E*_LUMO_ are −5.68/−3.78 eV (Table 1 and Fig. 1c). Compared with PG-IT, the HOMO and LUMO energy levels of PG-IT2F were slightly down-shifted, which is caused by the electron-withdrawing effect of difluorene substituents on the thiophene π-bridges in PG-IT2F.

Electron mobilities of the PSMAs were measured by the space charge limited current (SCLC) method, as shown in Supplementary Table 1. The electron mobility (*µ*_e_) of PY-IT2F (7.75 × 10^−4^ cm^2^ V^−1^ s^−1^) is higher than that of PY-IT (6.68 × 10^−4^ cm^2^ V^−1^ s^−1^), indicating that the fluorinated π-bridge effectively improved electron transport. The *µ*_e_ of PG-IT was calculated to be 5.93 × 10^−4^ cm^2^ V^−1^ s^−1^ (Supplementary Fig. 16), which is lower than that of PY-IT due probably to that the branched alkyl chain hinders the efficient molecular packing. The PG-IT2F film exhibits a higher *µ*_e_ (6.85 × 10^−4^ cm^2^ V^−1^ s^−1^) than that of PG-IT film, which further confirms that the difluorothiophene linker is beneficial to charge transport of the PSMAs.

To understand why the fluorinated π-bridge linker can improve electron transport of the PSMAs, we carried out the density functional theory (DFT) calculation to investigate the geometric and electronic properties of PG-IT and PG-IT2F. To simplify the calculations, we simulated the dimers with shorter alkyl side chains in representative of the two polymers. As shown in Supplementary Fig. 17a, PG-IT2F exhibits smaller dihedral angles than PG-IT, which indicates that the molecular conformation of PG-IT2F tends to be more planar. In addition, the difluorinated π-bridge linker promotes the delocalization of HOMO as well as LUMO over the entire molecule (Supplementary Fig. 17b), which may result in the higher electron mobility of PG-IT2F mentioned above.

### Photovoltaic performance of the PSMAs

**Fig. 2:**
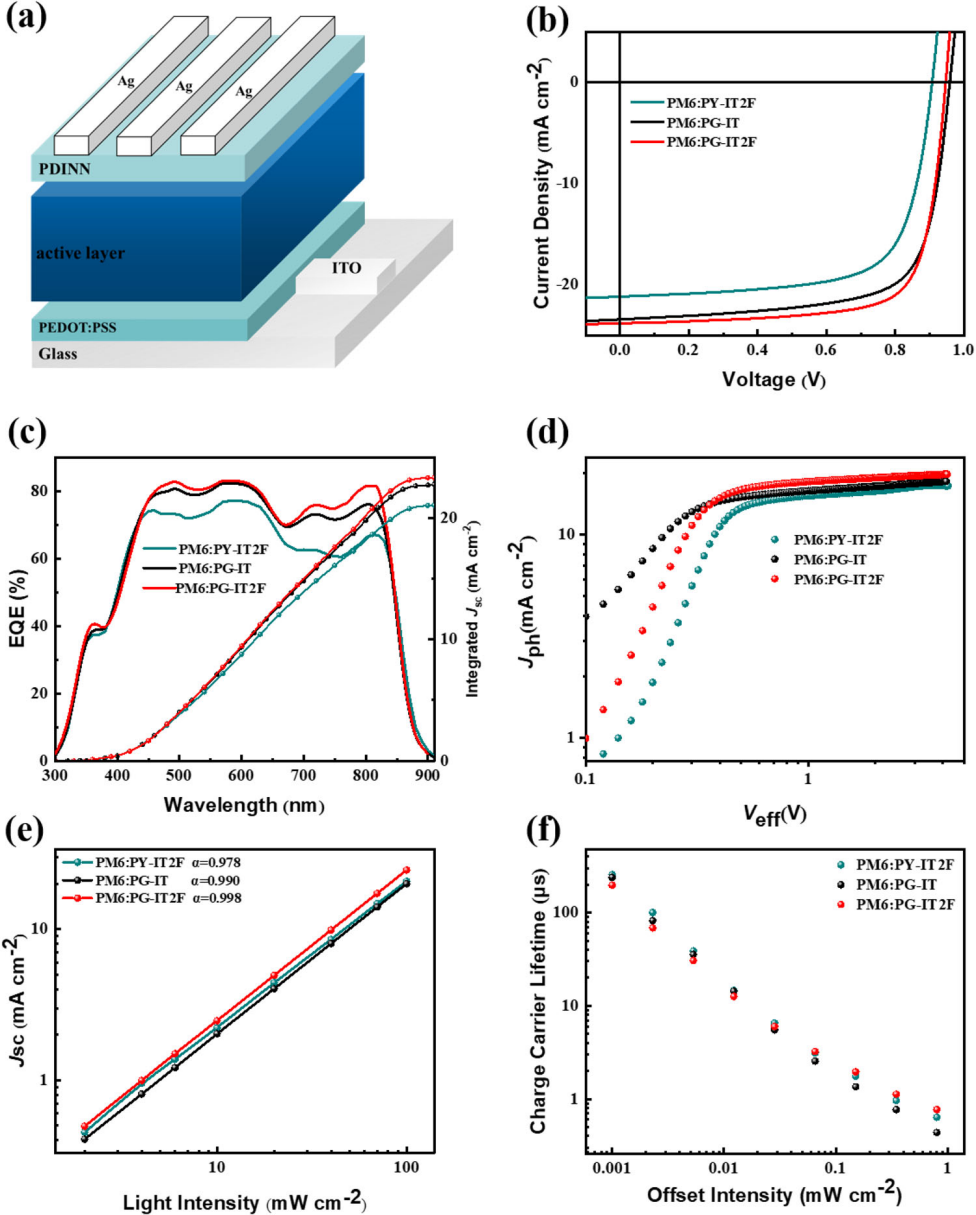
Device structure and photovoltaic performance of the all-PSC. **a** Device structure of the all-PSCs. **b** *J-V* curves of the optimized all-PSCs based on PM6:PSMAs under the illumination of AM1.5 G, 100 mW cm^−2^. **c** EQE curves of the corresponding all-PSCs. **d** *J*_ph_ versus *V*_eff_ of the all-PSCs. **e** Light intensity dependence of *J*_sc_ values of the corresponding all-PSCs. **f** Carrier lifetime curves under different light intensity obtained from TPV measurement. Source data are provided as a Source Data file.

To characterize the photovoltaic performance of the PSMAs, all-PSCs were fabricated with a conventional device structure of ITO/PEDOT: PSS/PM6: PSMAs/aliphatic amine-functionalized perylene-diimide (PDINN)^37^/Ag (Fig. 2a). To optimize the photovoltaic performance of the polymer acceptors, various device fabrication conditions were carefully screened, and the experimental results were summarized in Supplementary Tables 2–4. Figure 2b shows the current density-voltage (*J-V*) characteristics of the optimized all-PSCs, and their photovoltaic parameters were listed in Table 2 for a clear comparison. The optimal all-PSC based on PM6: PY-IT2F exhibits a moderate PCE of 14.11% with a *V*_oc_ of 0.91 V, *J*_sc_ of 22.18 mA cm^−2^ and a relatively lower FF of 69.98%, which is lower than that of the PY-IT-based devices^15^. Obviously, only a higher electron mobility is not enough to improve the photovoltaic performance of the PMSAs. The PM6:PG-IT based device shows a PCE of 16.09%, with a *V*_oc_ of 0.96 V, *J*_sc_ of 22.78 mA cm^−2^ and an FF of 71.43%, which is higher than that of the PY-IT2F based devices. Importantly, the all-PSC based on PM6: PG-IT2F demonstrated a champion PCE of 17.24%, with a *V*_oc_ of 0.95 V, *J*_sc_ of 24.03 mA cm^−2^ and an excellent FF of 75.46%, which is one of the highest reported PCE among the binary all-PSCs. The slightly reduced *V*_oc_ of the PG-IT2F-based devices than that of the PG-IT-based ones is mainly due to the lower-lying LUMO energy level of PG-IT2F caused by the introduction of fluorine substitution. Meanwhile, thanks to the smallest energy loss (*E*_loss_) of the PG-IT2F based devices (Supplementary Fig. 18 and Supplementary Table 5), the decrease of the *V*_oc_ from the PG-IT (0.96 V) to PG-IT2F based devices (0.95 V) is smaller than that from the PY-IT (0.93 V)^17^ to PT-IT2F based devices (0.91 V). From the external quantum efficiency (EQE) spectra of the optimal devices (Fig. 2c), it can be seen that all the devices show strong photo-response in the wavelength range of 430–860 nm, and the maximum EQE value of the PM6:PG-IT2F-based device exceeds 85%. The *J*_sc_ value of the PM6:PG-IT2F-based device integrated from the EQE spectrum is 23.32 mA cm^−2^, which is quite close to the *J*_sc_ values measured from the *J-V* curves, indicating the reliability of the photovoltaic performance measurements.

**Table 2:** Photovoltaic parameters of the optimal all-PSCs based on PM6:PSMAs (1:1, w/w), measured under the illumination of AM1.5 G, 100 mW cm^−2^

Active layers	*V*_oc_ (V)	*J*_sc_ (mA cm^−2^)	*J*_cal_^a^ (mA cm^−2^)	FF (%)	PCE (%)
PM6:PY-IT2F^b^	0.91	22.18	21.24	69.98	14.11
	(0.90 ± 0.003)	(21.96 ± 0.32)		(68.82 ± 0.93)	(13.91 ± 0.28)
PM6:PG-IT^b^	0.96 (0.96 ± 0.002)	23.46 (23.33 ± 0.28)	22.78	71.43 (71.02 ± 0.53)	16.09 (15.82 ± 0.23)^b^
PM6:PG-IT2F^b^	0.95 (0.95 ± 0.003)	24.03 (23.62 ± 0.24)	23.32	75.46 (74.97 ± 0.46)	17.24 (17.02 ± 0.32)

^a^Integrated from EQE values.

^b^Average values obtained from more than 10 different devices.

In addition, we investigated the stability of the all-PSCs based on PM6:PG-IT2F under continuous light irradiation of AM1.5 G, 100 mW cm^−2^ in a nitrogen-filled glovebox, in comparison with the device based on PM6:L8-BO where L8-BO^35^ is the corresponding SMA of the PSMA PG-IT2F. As shown in Supplementary Fig. 19, the PG-IT2F-based all-PSCs maintained a higher normalized PCE (retaining 94% of its initial PCE) in comparison with the SMA-based devices which retained 92.0% of its initial PCE, after the continuous light irradiation for 500 hours. The improvement in stabilities of the all-PSCs in comparison with the SMA-based device should be attributed to the higher illumination and morphology stability of the all-PSCs.

To understand the improved *J*_sc_ and FF of the all-PSCs based on the polymer acceptors from PY-IT2F, PG-IT to PG-IT2F, the hole (*µ*_h_) and electron (*µ*_e_) mobilities of the three polymer active layers blended with PM6 polymer donor were characterized by the SCLC method (Supplementary Fig. 20), and the results are listed in Supplementary Table 1. The *µ*_h_/*µ*_e_ values of the PM6:PY-IT2F blend film are 7.93 × 10^−4^/6.30 × 10^−4^ cm^2^ V^−1^ s^−1^. In comparison, the PM6:PG-IT blend film displays a similar *µ*_h_ of 7.21 × 10^−4^ cm^2^ V^−1^ s^−1^ while a decreased *µ*_e_ of 4.79 × 10^−4^ cm^2^ V^−1^ s^−1^. In comparison with PM6:PG-IT, the electron mobility increased to 5.54 × 10^−4^ cm^2^ V^−1^ s^−1^ with a slightly higher *µ*_h_ of 7.54 × 10^−4^ cm^2^ V^−1^ s^−1^ for the PM6:PG-IT2F blend film, which is benefitted from the difluorinated π-bridge linker in PG-IT2F. Moreover, owing to the increased electron mobility, the PM6:PG-IT2F active layer demonstrates a more balanced *µ*_h_/*µ*_e_ ratio, which may facilitate the charge extraction and collection of the corresponding all-PSCs.

The exciton dissociation and charge recombination characteristics in the blend films were investigated to further explore the reason of the improvement of device performance of the PG-IT2F-based all-PSCs. The dependence of the photocurrent density (*J*_ph_) on the effective voltage (*V*_eff_) was measured and the results are shown in Fig. 2d. Generally, the exciton dissociation probabilities (*P*_diss_) of the devices based on these polymer acceptors were estimated by the ratio of *J*_ph_ to saturation photocurrent density (*J*_sat_). As shown in Supplementary Table 6, the values of *J*_ph_/*J*_sat_ of the all-PSCs based on PM6:PY-IT2F, PM6:PG-IT, and PM6:PG-IT2F were calculated to be 89.24%, 90.72% and 93.13%, respectively, indicating that the device based on PG-IT2F has the highest exciton dissociation probability. The dependence of *J*_sc_ on the illumination light intensity (*P*_light_) was also investigated to provide further insight into the charge carrier recombination mechanism^38^. Generally speaking, the dependence of *J*_sc_ on *P*_light_ follows the equation *J*_*sc*_ ∝ *P*_light_^*α*^. As shown in the plots of log *J*_*sc*_ *vs*. log *P*_light_ in Fig. 2e, the *α* values obtained from the slope of the plots are 0.978, 0.990, and 0.998 for the PY-IT2F, PG-IT, and PG-IT2F-based devices, respectively. The *α* value of 0.998 (very close to 1) for the all-PSC based on PM6:PC-IT2F indicates that there is less bimolecular recombination of the charge carriers in the device based on PG-IT2F.

To provide deeper insight into the charge carrier recombination behavior in the all-PSCs in working conditions under illumination, we carried out the transient photovoltage (TPV) measurements (see Fig. 2f). The charge carrier lifetime in the PG-IT2F-based device is longer than that of the PY-IT2F and PG-IT-based devices under the illumination of various light intensities. Under the illumination with the maximum light intensity, the carrier lifetime of the PG-IT2F-based device was estimated to be 0.769 μs, while the values of the PY-IT2F and PG-IT-based devices are 0.736 μs and only 0.436 μs, respectively. Obviously, in addition to improving the electron transport, the F atom substituents also play an important role in stabilizing the formed radical anions on the polymer backbone and in prolonging the carrier lifetime, which are beneficial for reducing the charge carrier recombination rate and achieving higher *J*_sc_ and FF ^39^.

**Fig. 3:**
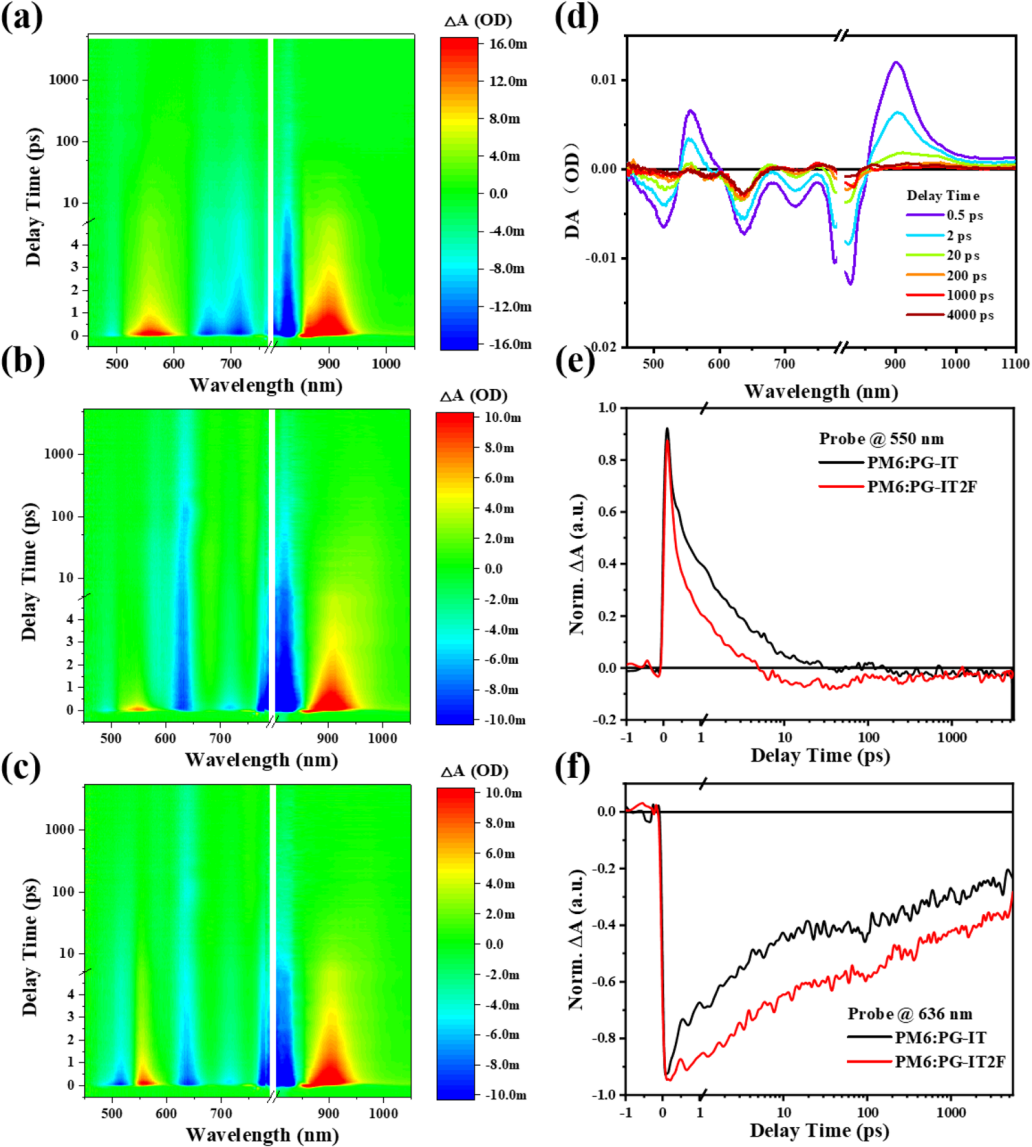
Femtosecond transient absorption kinetics. **a** PG-IT pristine film, **b** PM6:PG-IT blend film, and **c** PG-IT2F blend film with excitation at 840 nm. **d** Transient spectra of PM6:PG-IT blend at selective delay times. Transient kinetic traces probing at **e** 560 nm and **f** 636 nm for the PM6:PG-IT (black) and PM6:PG-IT2F (red) blend. Source data are provided as a Source Data file.

To further understand the charge transfer (CT) and recombination dynamics^40^ for the PSMAs-based system, femtosecond transient absorption spectroscopy (fs-TA) experiment was performed on the PG-IT and PG-IT2F systems. At first, we obtained the exciton spectrum of the polymer acceptor PG-IT by photoexciting its pristine film with a pump wavelength at 840 nm. Figure 3a shows that PG-IT exciton spectrum consists of three negative ground state bleach (GSB) peaks at 840 nm, 720 nm and 670 nm, and two excited state absorption (ESA) peaks at 560 nm and 900 nm. Then we investigated the CT behavior of the PM6:PG-IT blend by selectively exciting PG-IT in the blend at 840 nm. As shown in Fig. 3b, at early time, in addition to PG-IT exciton peaks, another GSB also appeared at 636 nm simultaneously, which is ascribed to the GSB of polymer donor PM6. This GSB is the result of ultrafast hole transfer from PG-IT exciton to PM6, generating the CT state in the blend. After 10 ps, exciton features at 560 nm and 720 nm decayed and the spectrum is dominated by a long-lived CT state which mainly includes PM6 donor GSB peak at 636 nm and PG-IT acceptor GSB peak at 840 nm. The fs-TA spectra of PM6:PG-IT2F blend were also measured under the same condition (Fig. 3c). The spectral profiles are similar for both blends, but their dynamics are quite different. We retrieved the kinetic traces of the blend films at 560 nm (Fig. 3e), which represent the ESA peaks of both PG-IT and PG-IT2F excitons. It is obvious that the PG-IT2F exciton decays faster than PG-IT in the blend, indicating a faster hole transfer channel in the PG-IT2F based devices. A faster hole transfer process could result in a higher CT yield, which can explain the higher *J*_sc_ in the PG-IT2F based devices. On the other hand, as shown in Fig. 3f, the GSB of PM6 at 636 nm decays slower in the PG-IT2F blend than that in the PG-IT blend, which means that the PM6:PG-IT2F blend exhibits a longer CT state lifetime. The relatively longer CT state lifetime suggests less charge recombination in the blend, contributing to higher *J*_sc_ and FF of the PG-IT2F based devices.

### Miscibility and morphology studies

**Fig. 4:**
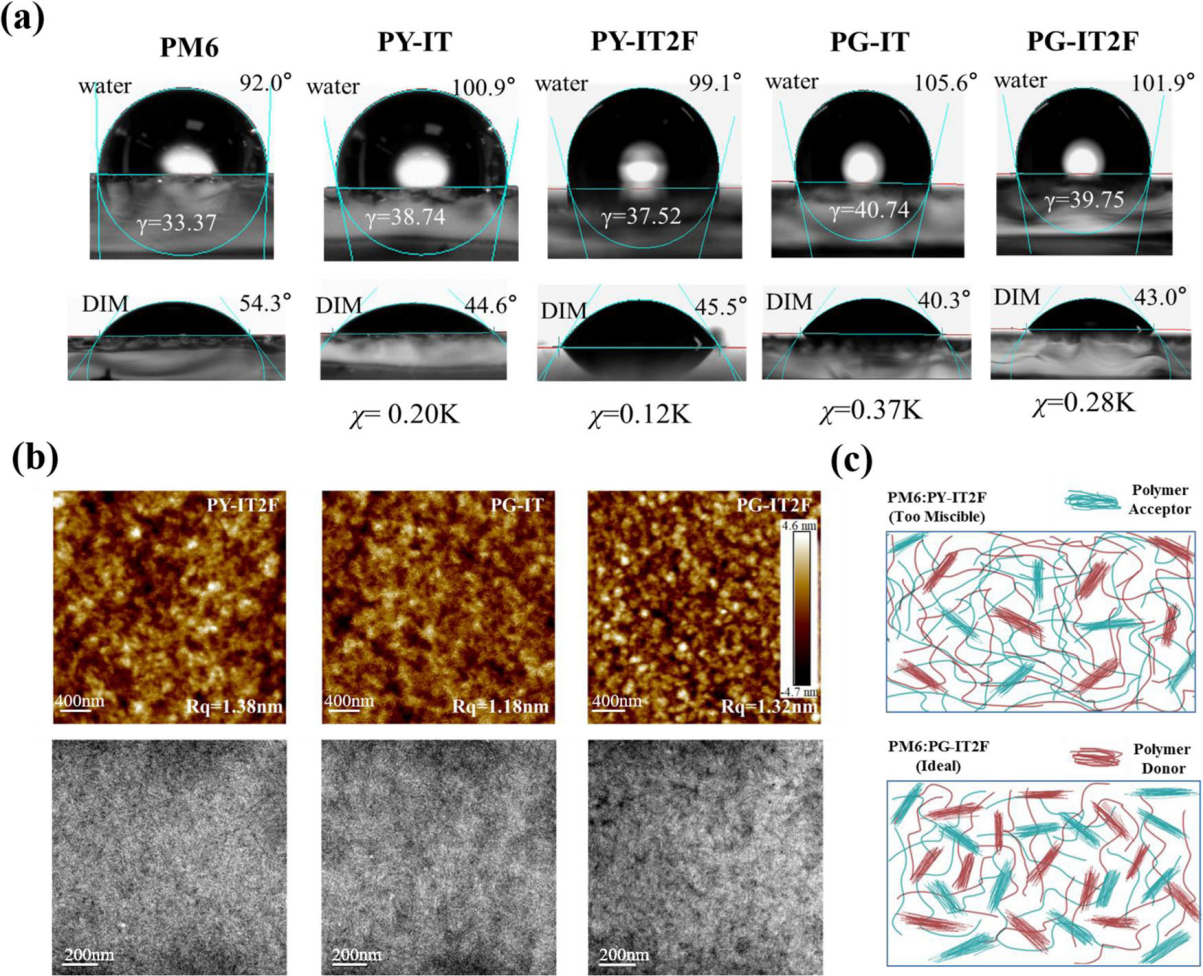
Contact angles and morphologies. **a** Contact angles of PM6, PY-IT, PY-IT2F, PG-IT, and PG-IT2F and the corresponding Flory-Huggins interaction parameters. **b** AFM height images and TEM images of PM6:PY-IT2F, PM6:PG-IT, and PM6:PG-IT2F blend film. **c** Schematic diagram of the morphology of PM6:PY-IT2F and PM6:PG-IT2F blend film.

To correlate the properties of all-PSCs with the miscibility between PM6 and the PSMAs. The Flory-Huggins interaction parameters (*χ*_da_) were calculated from contact angle measurements of the neat films. The corresponding surface energy of the polymer donor (*γ*_d_) and acceptors (*γ*_a_) (Fig. 4a) reveals information of the miscibility between the donor and acceptors, according to the equation

2 $${\chi }_{{da}}=K{(\sqrt{{\gamma }_{d}}-\sqrt{{\gamma }_{a}})}^{2}$$

where K is a constant. The *χ*_da_ value was calculated to be 0.12 K for the PM6:PY-IT2F blend films (Fig. 4a), which is too small in comparison with the other high performance systems^41^. The very small *χ* values correspond to strong D/A interactions and excessive miscibility of the donor and acceptor (Fig. 4c), which could raise the recombination losses^42^ and result in lower *J*_sc_ and FF of the all-PSCs. The *χ*_da_ value of the PM6:PG-IT blend films is 0.37 K, which is larger than that of PY-IT. This result indicates that the branched alkyl side chains does decrease the miscibility of the Y6-based polymer acceptors with PM6, which meets our original goal of adjusting the polymer miscibility by introducing branched alkyl chain. For PG-IT2F with the branched alkyl chain substituent, the excessive miscibility with PM6 was solved. Actually, the *χ*_da_ value of PM6:PG-IT2F is 0.28 K, an appropriate value for suitable (avoiding excessive) miscibility, which results in the higher exciton dissociation, lower charge recombination, and the improved photovoltaic performance of the corresponding all-PSCs.

In addition, atomic force microscopy (AFM) and transmission electron microscopy (TEM) were utilized to investigate the morphology of the active layers. As shown in Fig. 4b, the AFM images show that the blend films are smooth with phase separation increased from PM6:PY-IT2F to PM6:PG-IT2F. The TEM images of the PM6:PG-IT2F blend film demonstrate a more-defined phase separation of donor and acceptor with a bicontinuous interpenetrating network. These results are consistent with the results of contact angle measurements mentioned above.

**Fig. 5:**
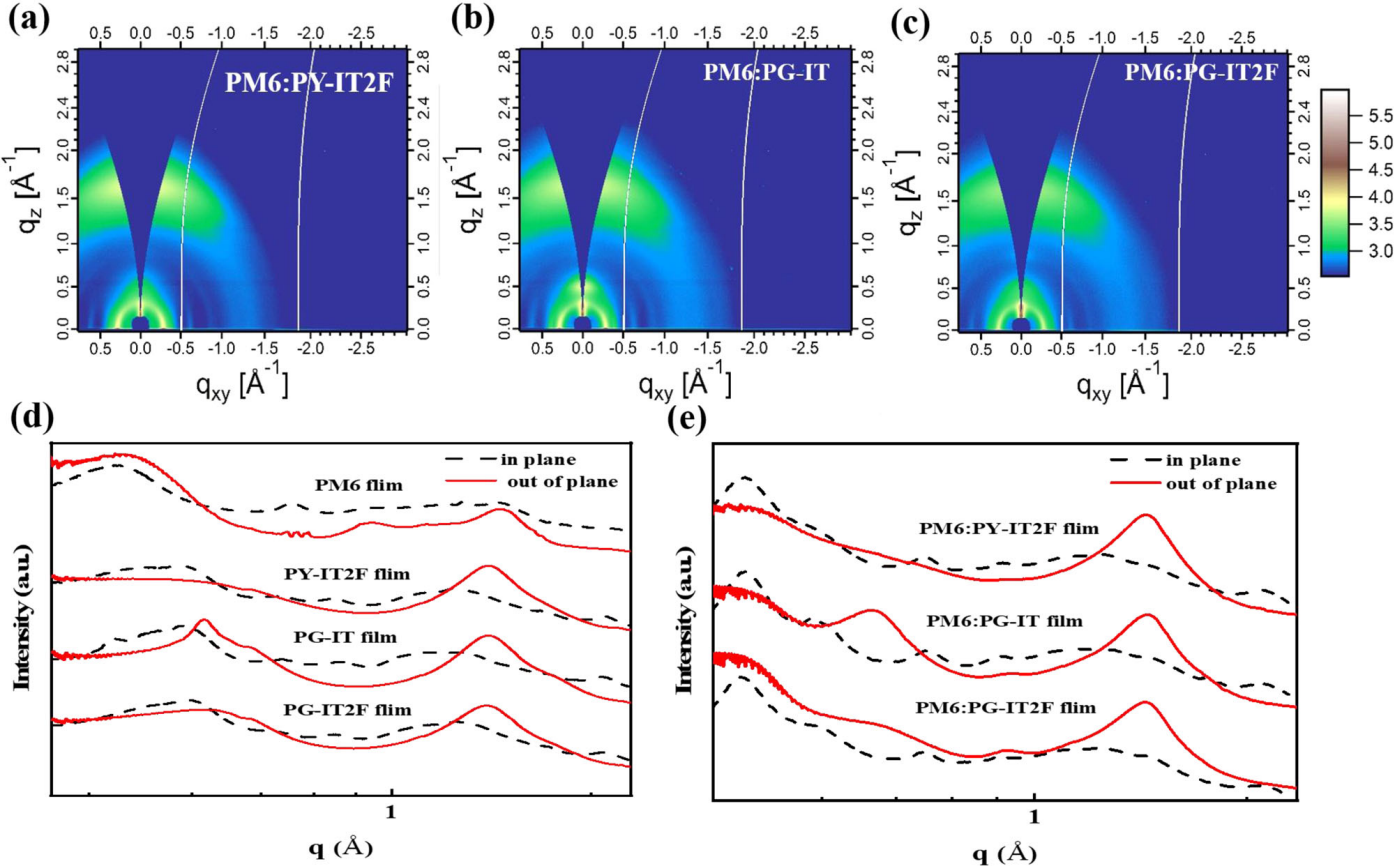
Results of GIWAXS measurements. **a** 2D GIWAXS patterns of the optimized blend films of PM6:PY-IT2F, **b** 2D GIWAXS patterns of the optimized blend films of PM6:PY-IT2F PM6:PG-IT, and **c** 2D GIWAXS patterns of the optimized blend films of PM6:PY-IT2F PM6:PG-IT2F. **d** Line cuts of GIWAXS images of the neat films. **e** Line cuts of GIWAXS images of the blend films. Source data are provided as a Source Data file.

Grazing incidence wide-angle X-ray scattering (GIWAXS) experiments were conducted to investigate the morphology features of the pristine and blend films (see Fig. 5 and Supplementary Fig. 21). As shown in Fig. 5d, the (010) peaks of the pristine PY-IT2F, PG-IT, and PG-IT2F films appear at *q*_z_ ≈ 1.56 Å^−1^, with similar π-π stacking distances (see Supplementary Fig. 16 and Supplementary Table 7). After blending with PM6, all the three blends exhibit predominant face-on orientation with the π-π (010) stacking peaks located at about 1.62 Å^−1^ in the out-of-plane (OOP) direction, and the lamellar (100) diffraction peaks in the in-plane (IP) direction at *q*_z_ ≈ 0.30 Å^−1^ (Fig. 5e), indicating that the face-on orientation is also dominant in the blends, which is beneficial for charge transport. It is worth mentioning that in the blend film, PG-IT still retains the characteristic peaks of its pristine film, and the peak in the OOP direction at *q*_z_ ≈ 0.50 Å^−1^ may be assigned as (001), which originates from the self-assembly of PG-IT^43^. For PY-IT2F, the π-π stacking peak in the blend films can be seen clearly in out-of-plane direction at 1.60 Å^−^^1^, with the crystalline coherence length (CCL) of 21.8 Å, which is the same as the neat film of PM6. Furthermore, the CCL values of (010) orientation are 23.5 and 20.8 Å for PG-IT and PG-IT2F neat films respectively. It is worth noting that after blending with PM6 polymer donor, the CCL of PG-IT in the blend film is significantly reduced from 23.5 Å to 19.7 Å. While there is little change in the CCL of PG-IT2F (from 20.8 Å to 20.5 Å), so that the PG-IT2F-based blend films exhibit slightly longer (010) CCL than that of PG-IT, and the PG-IT2F-based blend film with longer CCL also show better electron mobility. The different CCL changes of the two acceptors after blending with PM6 polymer donor may be due to their different miscibility with PM6.

In summary, the devices based on PY-IT2F obtain a relatively higher charge carrier mobility and a longer carrier lifetime owing to the introduction of F substituents on its thiophene linking unit, but its excessive miscibility with PM6 polymer donor leads to the more bimolecular recombination, which results in the lower *J*_sc_ and FF of the all-PSCs based on PY-IT2F. The devices based on PG-IT with branched alkyl upper side chains exhibit improved morphology and have moderate exciton dissociation and bimolecular recombination. However, the lowest charge carrier mobility and carrier lifetime limit the FF and PCE of the PG-IT-based devices. Compared with PY-IT2F and PG-IT, PG-IT2F with the branched alkyl upper side chains and difluorothiophene π-bridge linker has higher electron mobility, suitable miscibility with PM6 polymer donor and appropriate morphology of its active layer blending with PM6, leading to superior charge transfer performance, more balanced charge carrier mobility, longer CT state lifetime and decreased charge recombination, which results in the highest PCE with higher *J*_sc_, FF for the PG-IT2F-based all-PSCs.

## Discussion

A synergistic strategy of fluorinated thiophene π-bridge linker and branched alkyl upper side chain modification effectively improved the photovoltaic performance of the PSMAs. Three PY-IT derivatives of PY-IT2F, PG-IT, and PG-IT2F were synthesized by introducing difluorene substitution on the thiophene π-bridge linker and/or attaching branched alkyl upper side chains on the SMA building blocks of PY-IT. The PSMAs with the fluorinated π-bridge linker (PY-IT2F, PG-IT2F) possess increased electron mobility than those of the non-fluorinated PSMAs (PY-IT, PG-IT), which affords more balanced charge carrier transport with longer charge carrier lifetime in the active layer blending with PM6 polymer donor. While the excessive miscibility of PY-IT2F with PM6 results in poor photovoltaic performance of the PM6:PY-IT2F all-PSCs. By attaching the branched alkyl upper side chains, PG-IT2F affords optimal miscibility with PM6 and the PM6: PG-IT2F blend active layer showed good morphology with nanoscaled interpenetrating networks. Therefore, the all-PSCs based on PM6: PG-IT2F displayed efficient exciton dissociation and decreased charge recombination, thus demonstrated a high PCE of 17.24% with higher *J*_*sc*_ and FF. The PCE of 17.24% is one of the highest efficiency reported so far for the binary all-PSCs. This work indicates that the fluorination on π-bridge linker in conjunction with proper side chain modifications of the SMA building blocks is effective way to improve the photovoltaic performance of the PMSAs for realizing application of the all-PSCs.

## Methods

### Materials and synthesis

4,7-dibromo-5,6-dinitrobenzo[c][1,2,5]thiadiazole (97%), Stannane, tributyl[6-(2-butyloctyl)thieno[3,2-b]thien−2-yl] (95%) and 11-(bromomethyl)tricosane (98%) were purchased from Zhengzhou Alfa Chemical Co.,Ltd. Other common materials and chemical reagents were purchased from Innochem, J&K, Alfa Aesar and TCI Chemical Co. respectively. Toluene was distilled from sodium and benzophenone under nitrogen before using, and the reactions all performed under a nitrogen atmosphere. Polymer donor PM6 was purchased from Solarmer Materials Inc.

The synthetic routes of the polymer acceptors PY-IT2F, PG-IT, and PG-IT2F are shown in Supplementary Figs. 1–5. The detailed synthetic procedures and characterizations of the chemical structures of the monomers and the polymer acceptors are described in the Supplementary method section. The NMR spectra of the compounds are shown in Supplementary Figs. 6–11.

### Characterization of materials

^1^H NMR and ^13^C NMR spectra were recorded using a Bruker AV-400 spectrometer in a *d*–chloroform solution at 300 K, unless specified otherwise. Chemical shifts are reported as *δ* values (ppm) with tetramethylsilane as a benchmark. Mass spectra were measured on a Shimadzu spectrometer. GPC measurements are performed on Agilent PL-GPC 220 instrument with high-temperature chromatograph, using 1, 3, 5-Trichlorobenzene as the eluent at 150 °C. UV-Vis absorption spectra were recorded on the Hitachi U-3010 UV-vis spectrophotometer. For the measurement of solution absorption, PY-IT2F, PG-IT, PG-IT2F, and PM6 are dissolved in chloroform. For the film absorption measurements, PY-IT2F, PG-IT, PG-IT2F, and PM6 solutions in chloroform were spin-coated on quartz plates. The CV measurement was performed on the Zahner IM6e electrochemical workstation, using a glassy carbon electrode as the working electrode, platinum wire as the counter electrode, and Ag/AgCl as the reference electrode. The potential scanning speed is 50 mV s^−1^, and the electrolyte solution is 0.1 mol L^−1^ tetrabutylammonium hexafluorophosphate (Bu_4_NPF_6_) in anhydrous acetonitrile. The ferrocene/ferrocene (Fc/Fc^+^) redox pair was used as an internal reference.

### Fabrication and characterization of all-PSCs

The device structure of all-PSCs adopts the conventional structure nof ITO/PEDOT: PSS/active layer/PDINN/Ag. The pre-patterned ITO glass substrate (sheet resistance = 15 Ω sq^−1^) was sonicated in detergent, deionized water, acetone, and isopropanol, and subjected to 25 minutes in an ultraviolet ozone chamber (Jelight Company, USA) UV treatment. Filtering the PEDOT:PSS aqueous solution (Baytron P 4083, from HCStarck) through a 0.45 mm filter, and spin-coating it on the pre-cleaned ITO glass at 6000 rpm for 30 seconds, and then thermal annealing the ITO substrate with the PEDOT:PSS layer in the air at 150 °C for 0.5 h. The PM6: PSMAs (D:A = 1:1, 13.5 mg mL^−1^ in total) was dissolved in chloroform (CF) with adding 1-chloronaphthalene (CN) (1.5%, v/v) as solvent additive, and the blend solution was spin-coated with 3500 rpm for 30 s on the PEDOT:PSS layer, and then annealed at 100 °C for 5 minutes. Then PDINN methanol solution with a concentration of 1.0 mg mL^−1^ was deposited on the active layer at a spin-speed of 5000 rpm for 30 seconds to provide a PDINN cathode modification layer. After cooling to room temperature, the sample was transferred to the evaporation chamber. Under the vacuum condition of 1 × 10^−5^ Pa, ~100 nm of Ag was evaporated as the top electrode. The device area is 6.0 mm^2^.

The *J-V* characteristics of the all-PSCs are measured in a nitrogen glove box equipped with a Keithley 2450 Source Measure device. Using Oriel Sol3A Class AAA Solar Simulator (model, Newport 94023 A) with 450 W xenon lamp and air quality (AM) 1.5 filter as the light source. The light intensity is calibrated to 100 mW cm^−2^ by Newport Oriel 91150 V reference battery. The EQE value is measured by the solar cell spectral response measurement system QE-R3-011 (Taiwan Enli Technology Co., Ltd.). Standard single-crystal silicon photovoltaic cells are used to calibrate the light intensity of each wavelength. Similarly, in the TPV measurements, the fabrication of the device adopts the above methods. The data were obtained by the all-in-one characterization platform, Paios (Fluxim AG, Switzerland). In the TPV measurement, the light intensity is 0.10%, 0.23%, 0.53%, 1.23%, 2.83%, 6.52%, 15.0%, 34.6%, and 80.0%, respectively, relative intensity is 20.0% and settling time is 30.0 ms, pulse length is 5.0 ms and the follow-up time is 30.0 µs.

### Measurement of charge carrier mobilities

The device structure of ITO/PEDOT: PSS/active layer/MoO_3_/Ag is used to test hole mobility, and ITO/ZnO/active layer/PDINN/Ag device structure is used to test electron mobility. The hole and electron mobilities are calculated according to the SCLC method equation:

3 $$J=9{\mu {{{{{\rm{\varepsilon }}}}}}}_{r}{{{{{{\rm{\varepsilon }}}}}}}_{0}{V}^{2}/8{d}^{3}$$

where *J* is the current density, *µ* is the hole or electron mobility, *V* is the internal voltage in the device, *ε*_r_ is the relative dielectric constant of active layer material, *ε*_0_ is the permittivity of empty space, and *d* is the thickness of the active layer.

### Transient absorption spectroscopy

Femtosecond transient absorption spectrometer is composed of a regenerative-amplified Ti: sapphire laser system (Coherent) and Helios pump-probe system (Ultrafast Systems). The regenerative-amplified Ti: sapphire laser system (Legend Elite-1K-HE, center wavelength of 800 nm, pulse duration of 25 fs, pulse energy of 4 mJ, repetition rate of 1 kHz) was seeded with a mode-locked Ti: sapphire laser system (Vitara) and pumped with a Nd: YLF laser (Evolution 30). The output 800 nm fundamental of the amplifier was split into two beam pulses. The main part of the fundamental beam went through the optical parametric amplifiers (TOPAS-C), whose output light was set as the pump light with wavelength of 840 nm and chopped by a mechanical chopper operating at frequency of 500 Hz. A small part of the fundamental beam was introduced into the TA spectrometer in order to generate the probe light. After passing through a motorized optical delay line, the fundamental beam was focused on a sapphire crystal or YAG crystal, which was used to generate the white-light continuum (WLC) probe pulses with wavelength of 430 to 820 nm or 800 to 1400 nm, respectively. The optical path difference between the pump light and the probe light, which is controlled by the motorized optical delay line, was used to monitor the transient states at different pump-probe delay. A reference beam was split from the WLC in order to correct the pulse-to-pulse fluctuation of the WLC. The pump was spatially and temporally overlapped with the probe beam on the sample. Excitation energy of the pump pulse was set to 2 μJ/cm^2^ to avoid singlet-singlet annihilation. The film samples for TA measurements were prepared by spin-coating the corresponding materials on thin quartz plates. The film samples were thermally annealed in the same way as the actual device.

### Energy loss measurements

Fourier-transform photocurrent spectroscopy external quantum efficiency (FTPS-EQE) was measured by using an integrated system (PECT-600, Enlitech). External electroluminescence quantum efficiency (EQE_EL_) measurements were performed by applying external voltage/current sources through the devices (REPS, Enlitech). The total *E*_loss_ (Δ*E*) can be separated into three parts:

4 $$\Delta E	={E}_{g}-{{qV}}_{{OC}}=\left({E}_{g}-{{qV}}_{{OC}}^{{SQ}}\right)+\left({{qV}}_{{OC}}^{{SQ}}-{{qV}}_{{OC}}^{{rad}}\right)+\left({{qV}}_{{OC}}^{{rad}}-{{qV}}_{{OC}}\right)\\ 	=\left({E}_{g}-{{qV}}_{{OC}}^{{SQ}}\right)+{{qV}}_{{OC}}^{{rad},{belowgap}}+{qV}_{{OC}}^{{non}-{rad}}={\Delta E}_{1}+{\Delta E}_{2}+{\Delta E}_{3}$$

Among them, Δ*E*_1_ (${E}_{g}-{{qV}}_{{oc}}^{{SQ}}$), from the radiative recombination loss above the *E*_g_, is unavoidable in all types of solar cells. The second part, Δ*E*_2_ (${{qV}}_{{oc}}^{{SQ}}-{{qV}}_{{oc}}^{{rad}}={q\Delta {{{{{\rm{V}}}}}}}_{{oc}}^{{rad}}$), stems from the radiative recombination loss below the bandgap, where the ${\Delta V}_{{oc}}^{{rad}}$ can be calculated by realistic radiative recombination using a reciprocity relation between FTPS-EQE and EQE_EL_. According to the estimated equation, the third part, Δ*E*_3_ = −*kTln*(*EQE*)_*EL*_.

### AFM, TEM, and contact angle measurements

The morphologies of the blend films were investigated by AFM (Agilent Technologies, 5500 AFM/SPM System, USA) in contacting mode with a 5 μm scanner. TEM measurements were performed in a JEM−2100F. Contact angle tests were implemented on the Drop Shape Analyzer (DSA100, KRÜSS) in static mode at room temperature. The surface free energy of each film was obtained by computer fitting calculations. In detail, the obtained contact angle calculated by averaging the left and right angles of a sessile drop was measured by KRÜSS software with the tangential method. The deionized water and diiodomethane (1.5 µL) were dropped on the Si/SiO_2_ wafers with the neat film and then the droplet was snapshotted after the equilibrium on the gas-liquid-solid interface. The contact angle needs to be fixed at the standard deviation of ±1°.

### GIWAXS characterization

GIWAXS measurements were performed at beamline 7.3.3 at the Advanced Light Source. Samples were prepared on Si substrates using identical blend solutions as those used in devices. The 10 keV X-ray beam was incident at a grazing angle of 0.13°, selected to maximize the scattering intensity from the samples. The scattered x-rays were detected using a Dectris Pilatus 2 M photon counting detector.

### Reporting summary

Further information on research design is available in the Nature Research Reporting Summary linked to this article.

## Supplementary information

Supplementary Information

Solar Cells Reporting Summary

## Data Availability

The data that support the findings of this study are presented in Supplementary Information. Source data are provided with this paper.

## Source data

Source Data
