# Supplementary material for: High performance polymerized small molecule acceptor by synergistic optimization on π-bridge linker and side chain
Source: Nat Commun. 2022 Sep 7;13:5267. doi: 10.1038/s41467-022-32964-z (PMC9452561; doi:10.1038/s41467-022-32964-z)
Supplement: Supplementary file 1 — Supplementary Information [file 41467_2022_32964_MOESM1_ESM.pdf]

Supplementary Information

High performance polymerized small molecule acceptor by synergistic optimization on π-bridge linker and side chain

Sun, *et al*.

Supplementary Figures

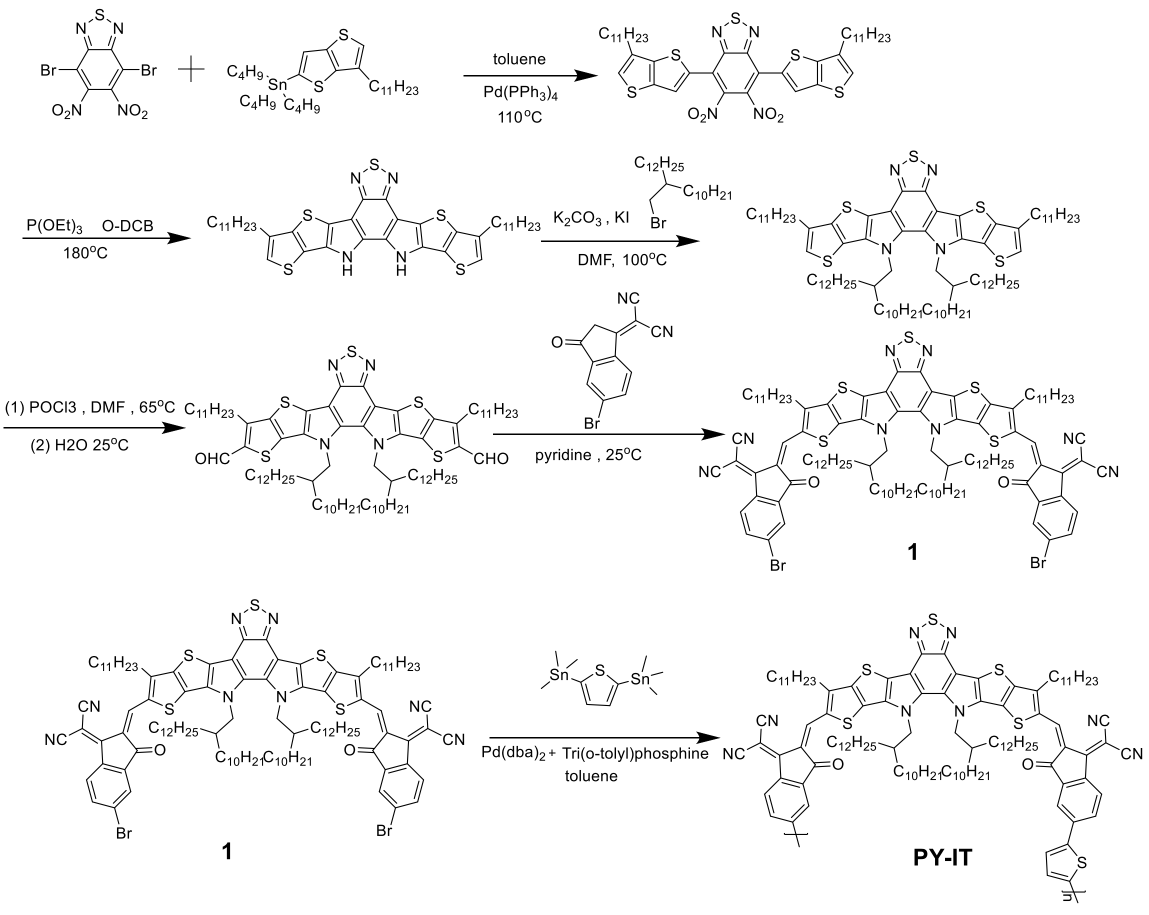

Supplementary Figure 1. Synthetic route of the SMA building block 1 and PY-IT.

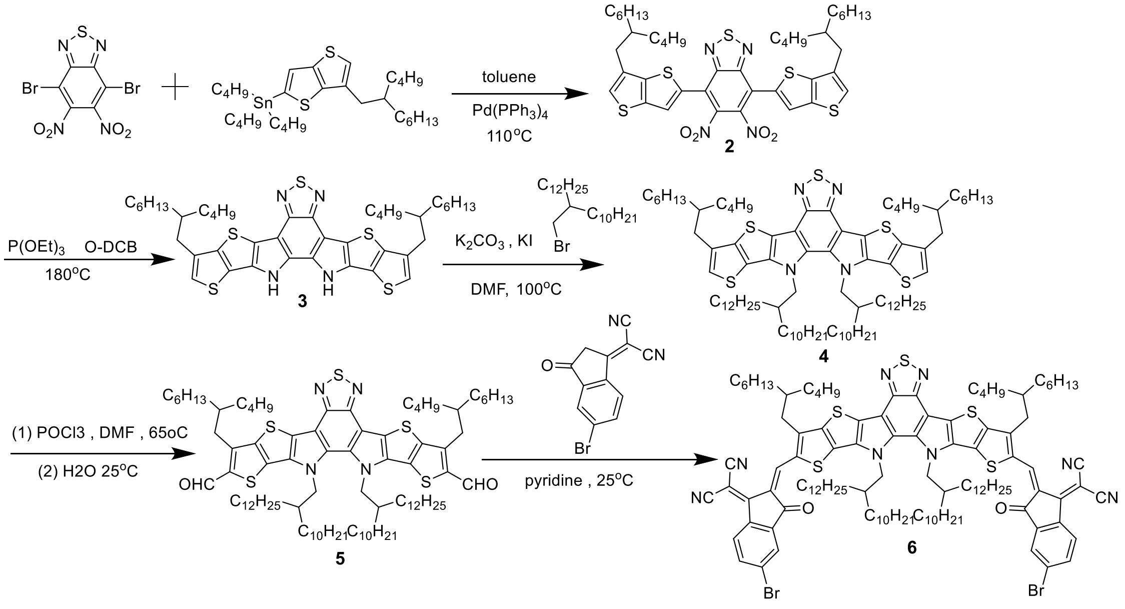

Supplementary Figure 2. Synthetic route of the branched SMA building block 6.

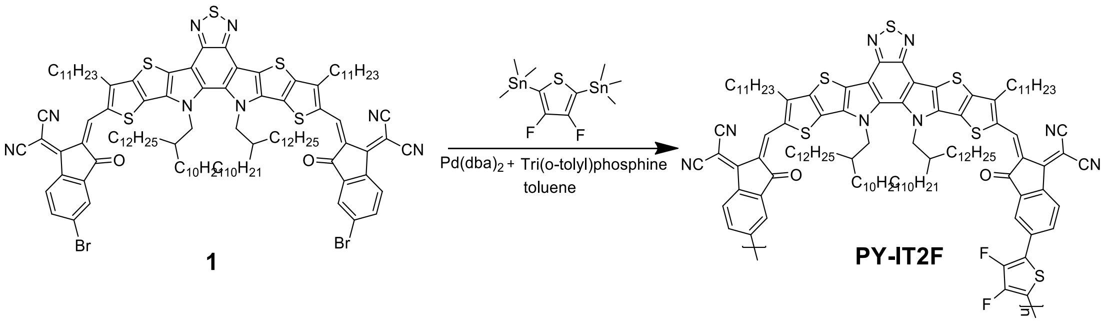

Supplementary Figure 3. Synthetic route of the PY-IT2F.

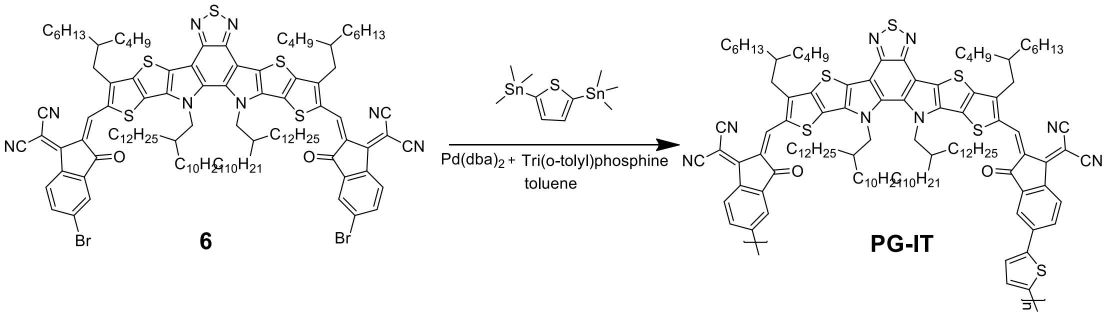

Supplementary Figure 4. Synthetic route of the PG-IT.

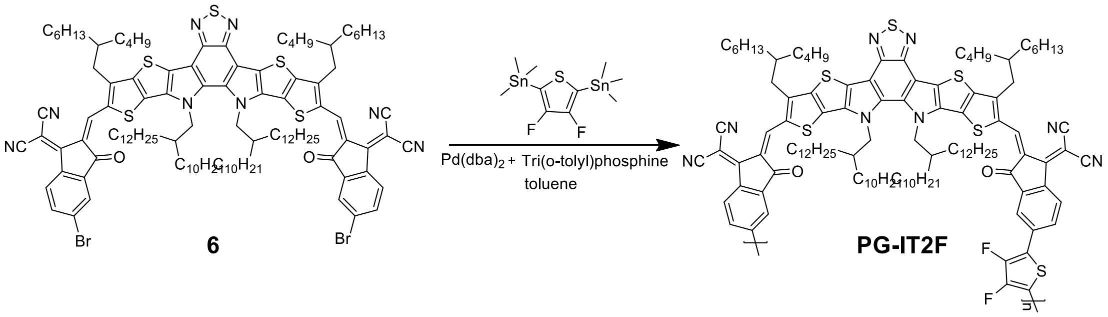

Supplementary Figure 5. Synthetic route of the PG-IT2F.

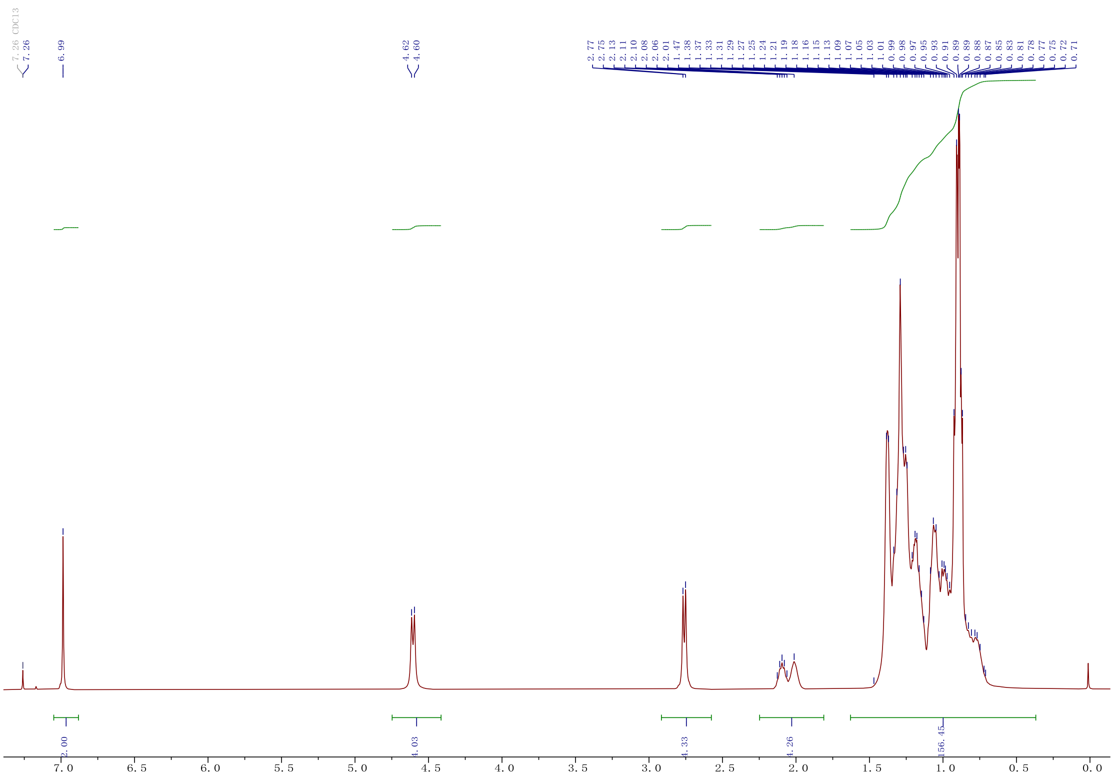

Supplementary Figure 6. ^{1}H NMR spectrum of intermediate **4** in $CDCl_3$

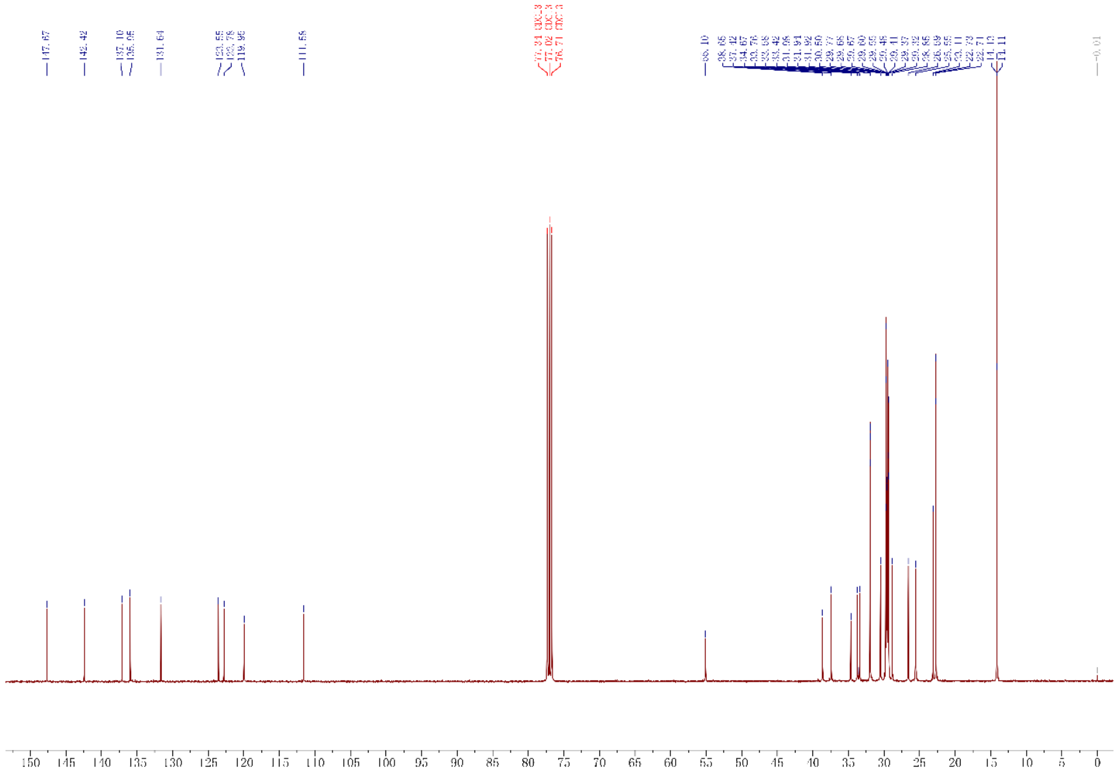

Supplementary Figure 7. ^{13}C NMR spectrum of intermediate **4** in $CDCl_3$

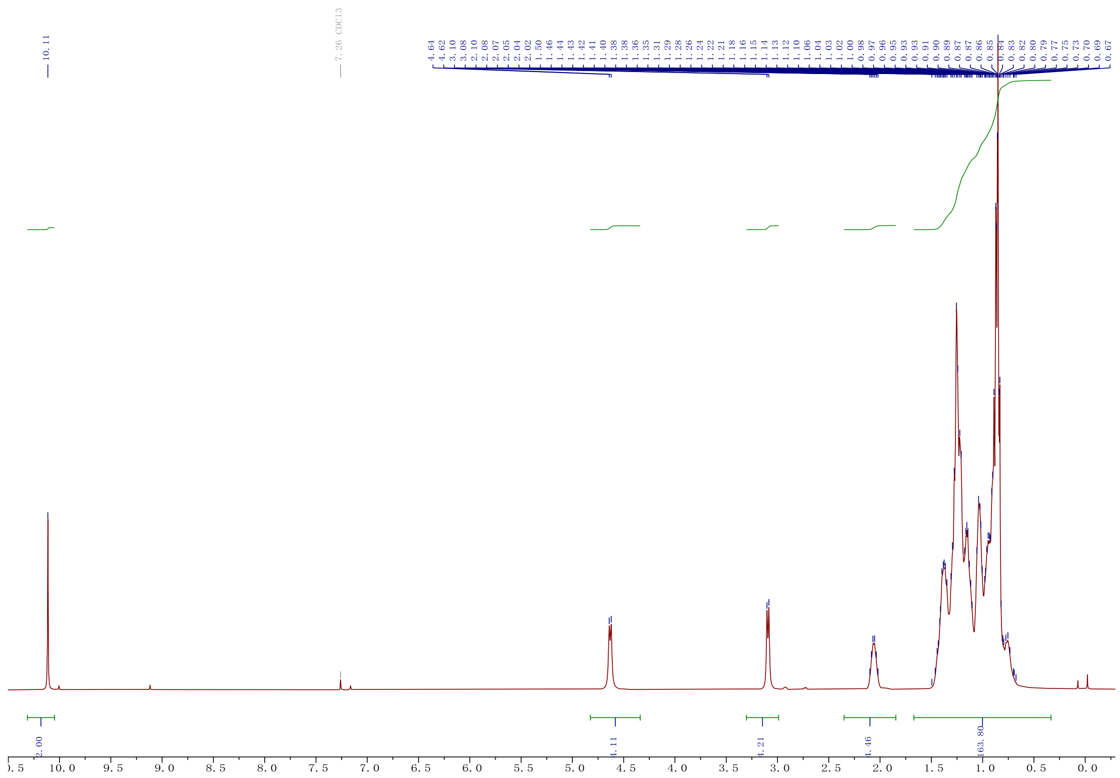

Supplementary Figure 8. ^{1}H NMR spectrum of intermediate **5** in $CDCl_3$

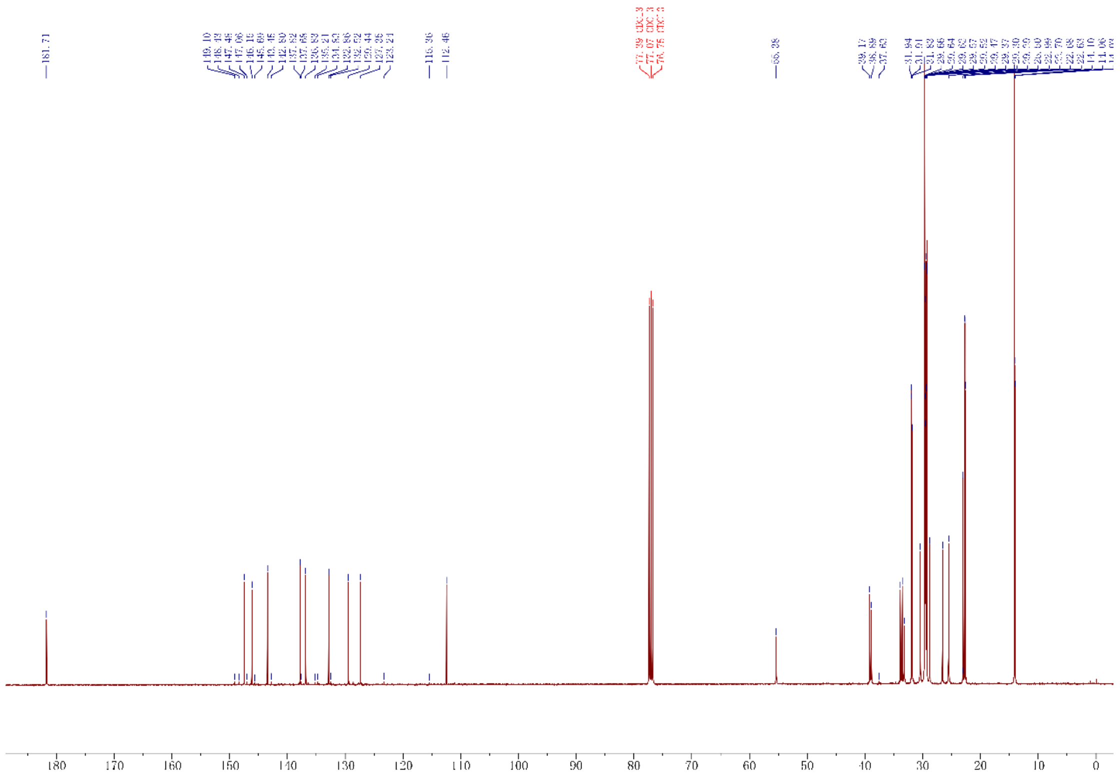

Supplementary Figure 9. ^{13}C NMR spectrum of intermediate **5** in $CDCl_3$

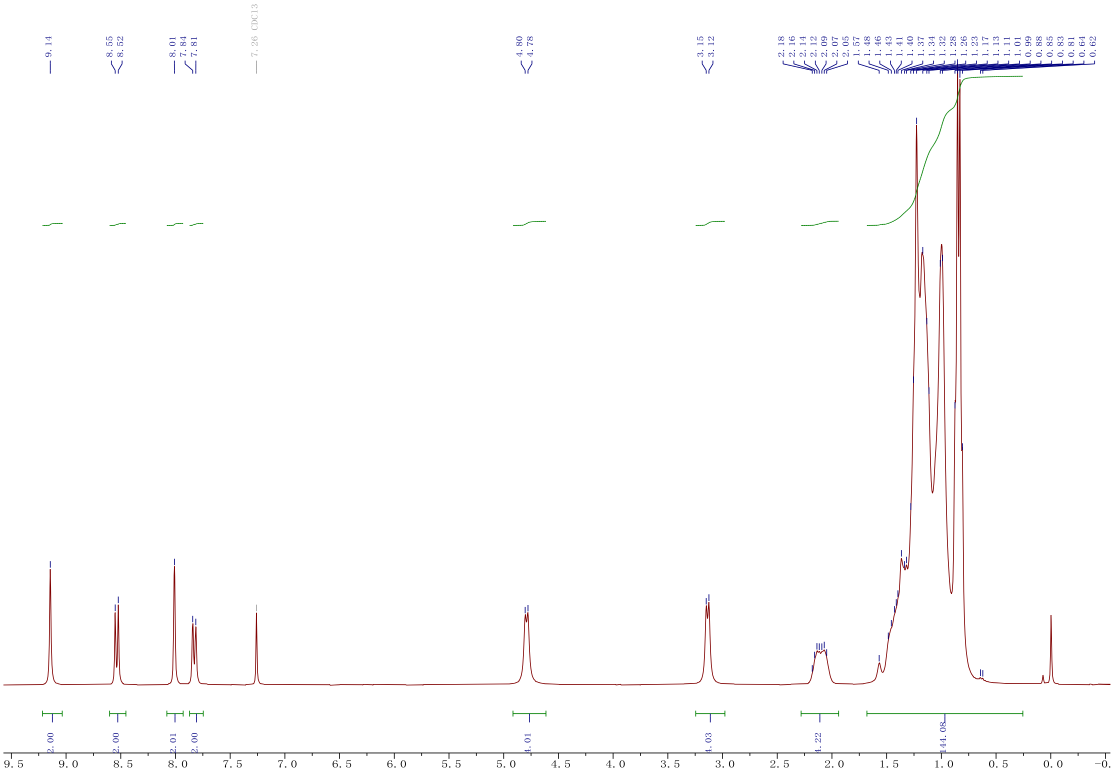

Supplementary Figure 10. ^{1}H NMR spectrum of monomer **6** in $CDCl_3$

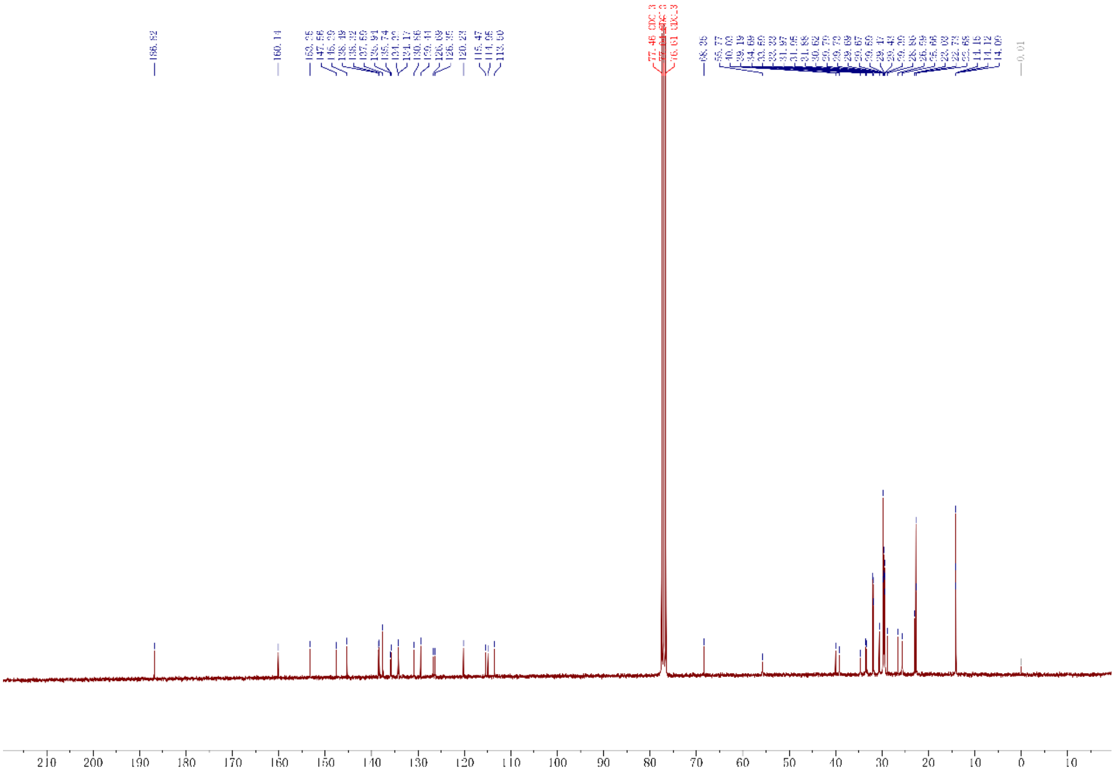

Supplementary Figure 11. ^{13}C NMR spectrum of monomer **6** in $CDCl_3$

acceptor	M_p	M_n	M_w	M_z	M_{z+1}	M_v	PDI
PY-IT2F	79164	26336	84740	169358	249640	73056	3.217
PG-IT	38034	11941	28556	54189	76847	25006	2.391
PG-IT2F	42976	17073	46744	90764	129039	40588	2.738

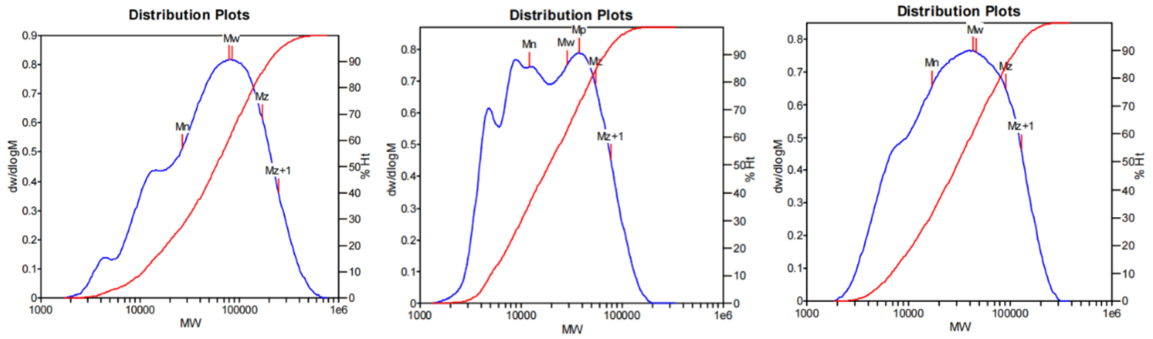

Supplementary Figure 12. GPC chart related to the molecular weight of PY-IT2F, PG-IT and PG-IT2F.

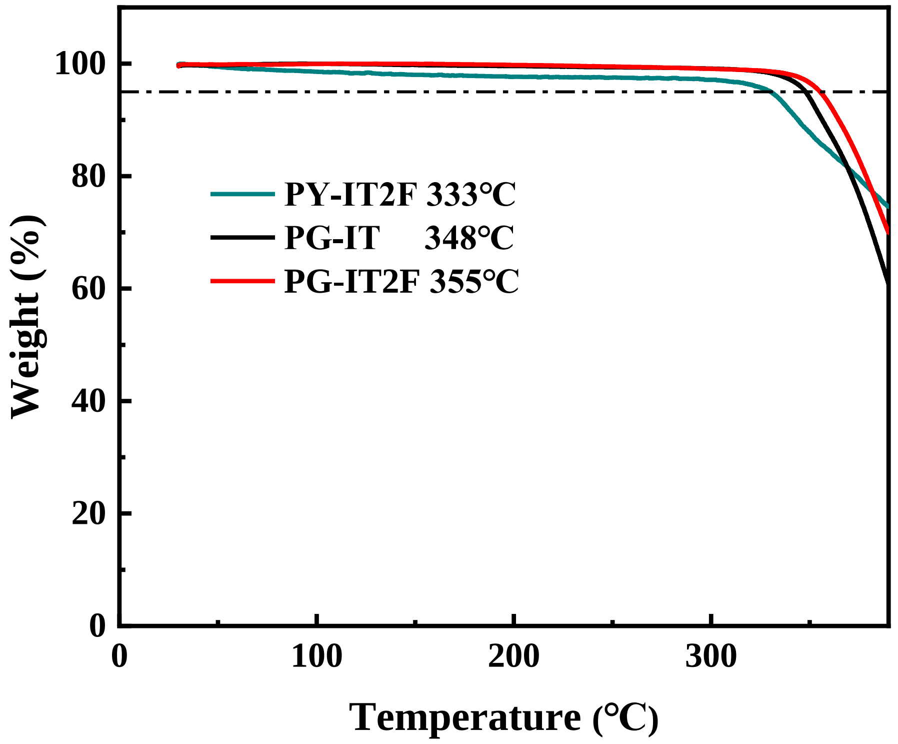

Supplementary Figure 13. TGA plots of PY-IT2F, PG-IT and PG-IT2F with a heating rate of 10 °C min^{-1} under N_2 atmosphere.

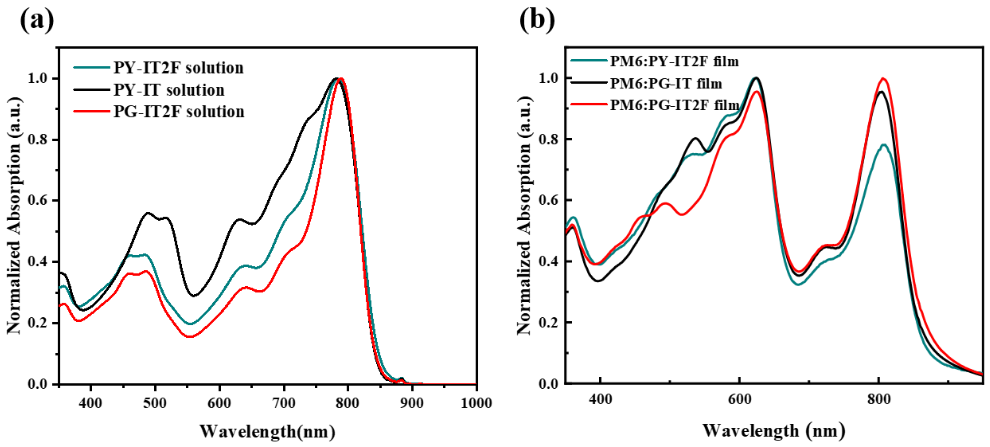

Supplementary Figure 14. UV-Vis absorption spectra: (a) Absorption spectra of the PSMAs in chloroform solution, (b) Absorption spectra of the polymer blend films of PM6: PSMAs.

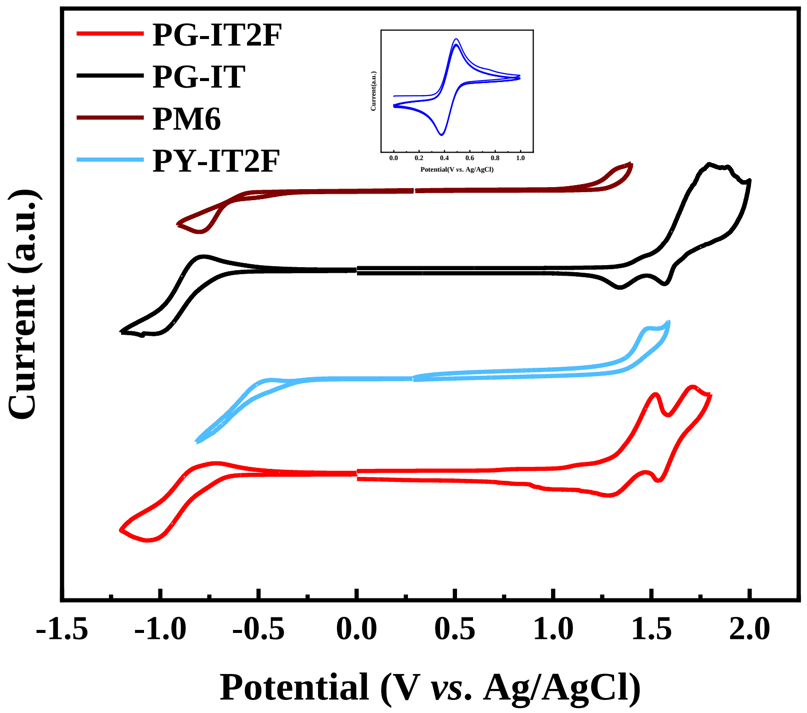

Supplementary Figure 15. Cyclic voltammograms of PM6, PY-IT2F, PG-IT and PG-IT2F films.

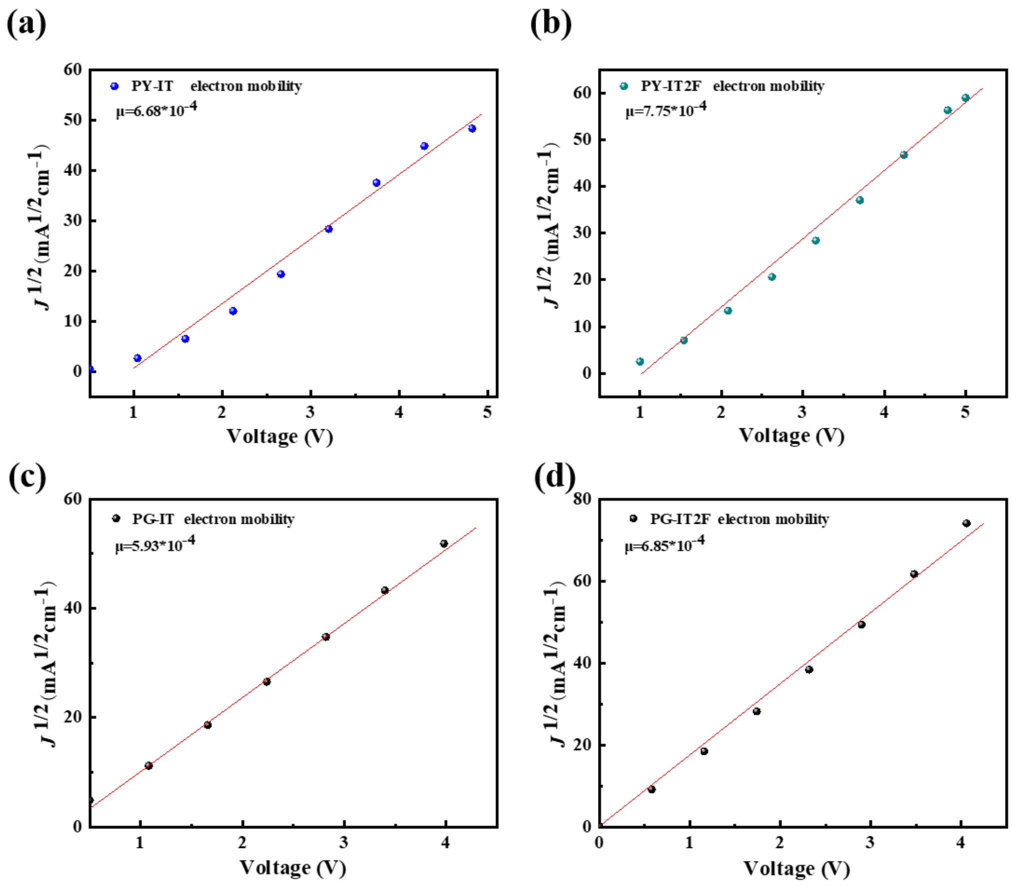

Supplementary Figure 16. **Electron mobility measurements of the PSMAs**: $J^{1/2}\sim V$ characteristics of the electron mobility measurement plots of the neat films of (a) PY-IT, (b) PY-IT2F, (c) PG-IT and (d) PG-IT2F in the dark.

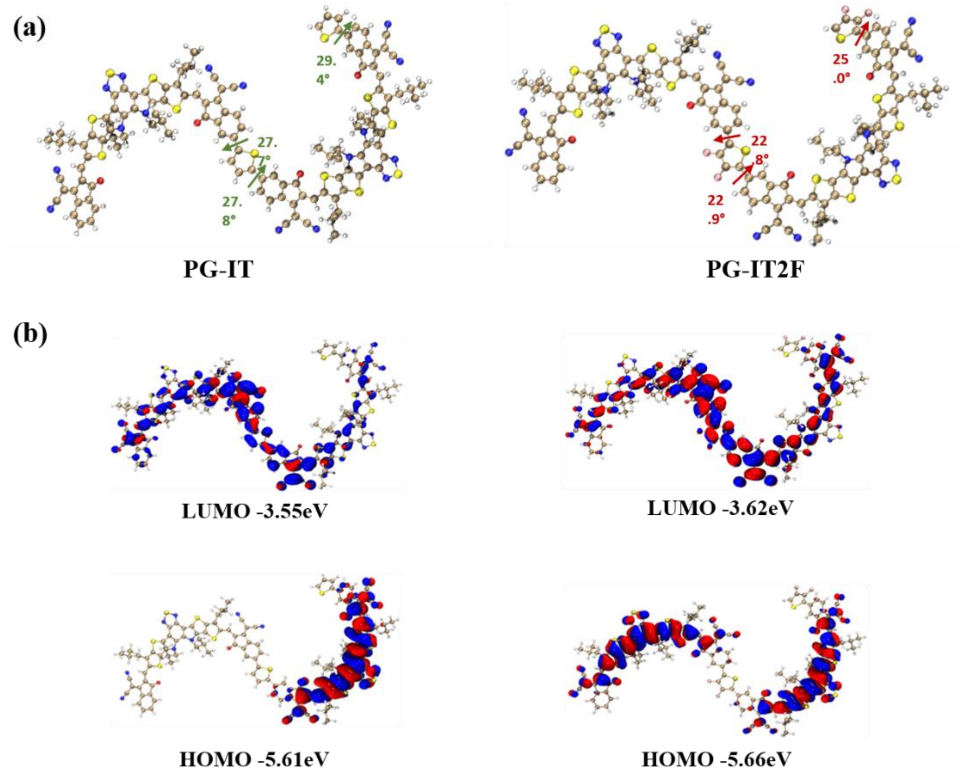

Supplementary Figure 17. Result of DFT calculation (a) Molecular configuration, dihedral angle of PG-IT and PG-IT2F and (b) Simulation of frontier orbital energy levels in PG-IT and PG-IT2F, calculated by DFT.

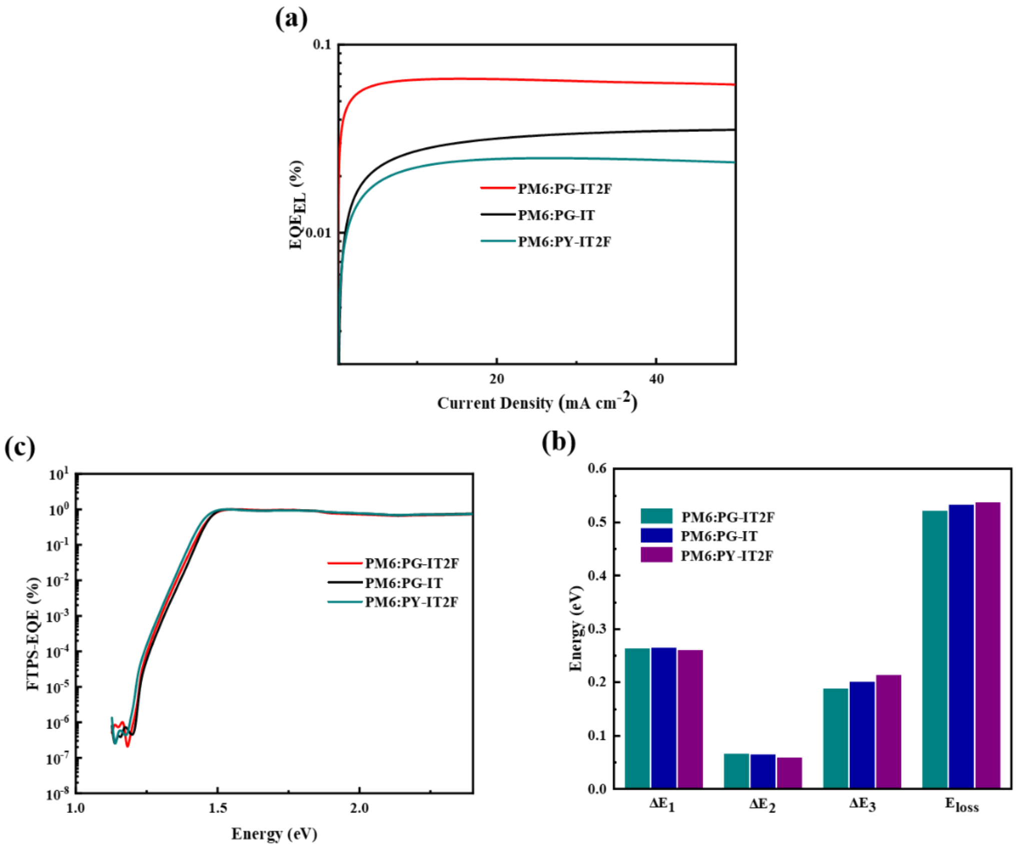

Supplementary Figure 18. **Energy loss measurements** (a) The EQE_{EL} of all-PSCs at various injection current densities. (b) FTPS-EQE spectra of three all-PSCs. (c) E_{loss} and its detailed three parts of ΔE_1, ΔE_2 and ΔE_3.

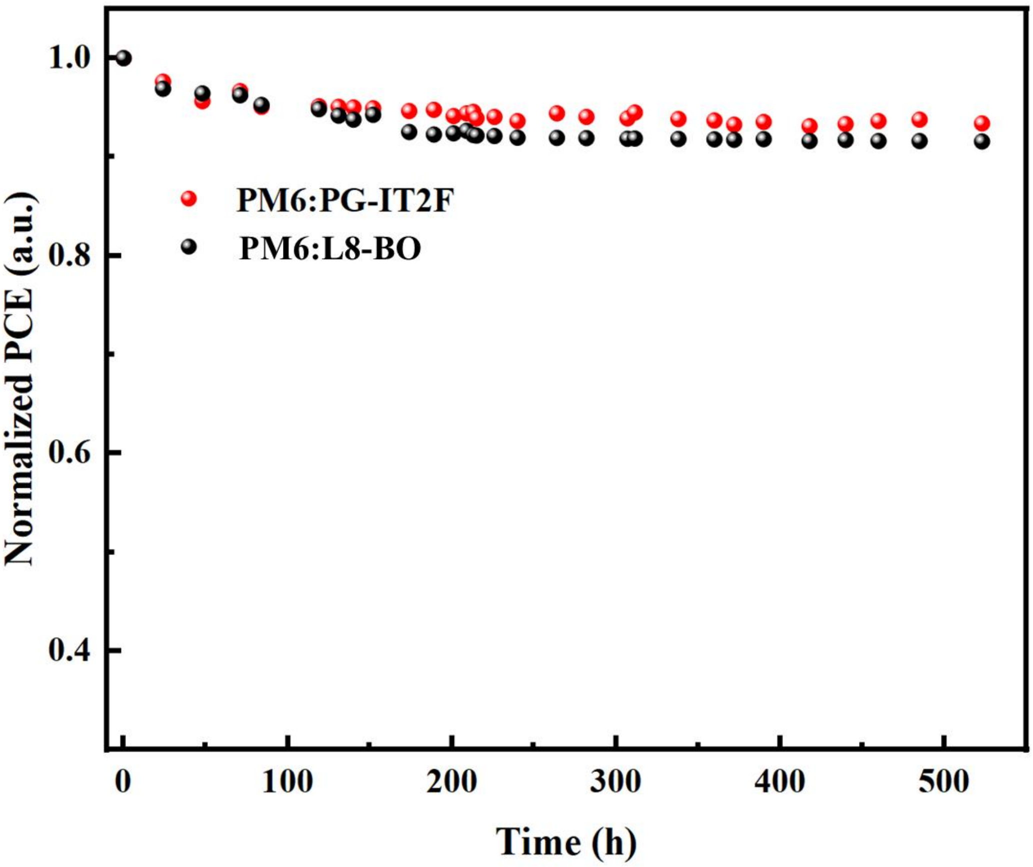

Supplementary Figure 19. Normalized PCEs stability of the devices under continuous illumination of AM1.5 G, 100 mW cm^{-2} in a nitrogen-filled glovebox.

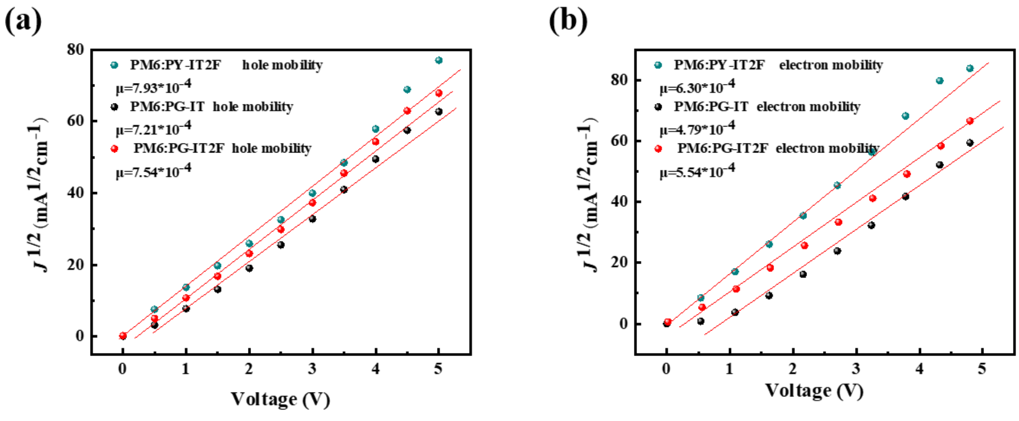

Supplementary Figure 20. Mobility measurements of blend films (a) $J^{1/2}$~V characteristics of the hole mobility measurement plots of the polymer blend active layers in the dark, (b) $J^{1/2}$~V characteristics of the electron mobility measurement plots of the polymer blend active layers in the dark.

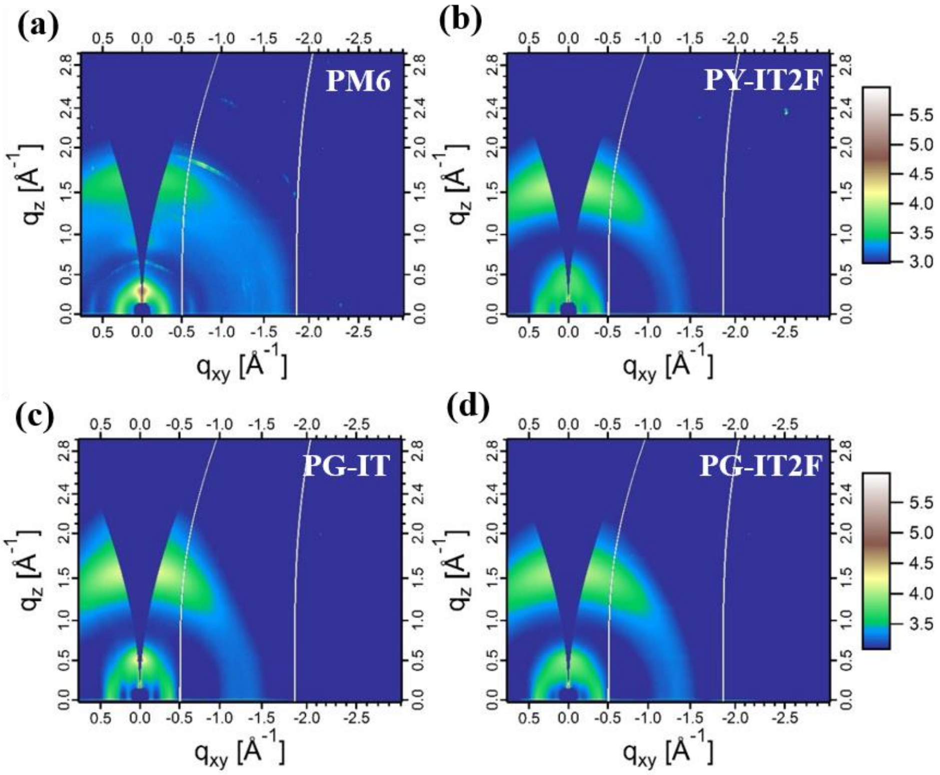

Supplementary Figure 21. 2D GIWAXS patterns of the neat films of (a) PM6, (b) PY-IT2F, (c) PG-IT, and (d) PG-IT2F.

Supplementary Tables

Supplementary Table 1. Detail data of the hole (μ_h) and electron (μ_e) mobilities.

Active layer	μ_h (× 10^{-4} $cm^2V^{-1}s^{-1}$)	μ_e (× 10^{-4} $cm^2V^{-1}s^{-1}$)	μ_h/μ_e
PM6:PY-IT2F	7.93	6.30	1.26
PM6:PG-IT	7.21	4.79	1.51
PM6:PG-IT2F	7.54	5.54	1.36

Supplementary Table 2. Photovoltaic performance parameters of the all-PSCs based on PM6:PG-IT2F, under the illumination of AM 1.5 G, 100 mWcm^{-2}.

D:A Weight Ratio	Concentration (CF)	Additive (CN) (%)	Annealing (℃)	V_{oc} (V)	J_{sc} (mA cm^{-2})	FF (%)	PCE (%)
1.2:1		w/o		0.957	20.88	61.24	12.23
1:1.2		w/o		0.951	20.62	58.46	11.46
		w/o		0.952	21.51	62.04	12.70
	13.5mg/ml	1	100	0.948	23.34	73.62	16.28
1:1		1.5		0.951	24.03	75.46	17.24
		2		0.948	23.52	73.19	16.32

Supplementary Table 3. Photovoltaic performance parameters of the all-PSCs based on PM6:PG-IT, under the illumination of AM 1.5 G, 100 mWcm^{-2}.

D:A Weight Ratio	Concentration (CF)	Additive (CN) (%)	Annealing (°C)	V_{oc} (V)	J_{sc} (mA cm^{-2})	FF (%)	PCE (%)
1.2:1	13.5mg/ml	w/o	100	0.964	20.47	60.77	11.99
1:1.2		w/o		0.961	20.32	58.46	11.42
1:1		w/o		0.963	21.13	62.04	12.61
		1.5		0.960	23.46	71.43	16.09

Supplementary Table 4. Photovoltaic performance parameters of the all-PSCs based on PM6:PY-IT2F, under the illumination of AM 1.5 G, 100 mWcm^{-2}.

D:A Weight Ratio	Concentration (CF)	Additive (CN) (%)	Annealing (°C)	V_{oc} (V)	J_{sc} (mA cm^{-2})	FF (%)	PCE (%)
1.2:1	13.5mg/ml	1.5	100	0.912	22.25	63.21	12.83
1:1.2		1.5		0.903	22.38	65.60	13.26
1:1		1.5		0.909	22.18	69.98	14.11

Supplementary Table 5. Detailed energy loss of PG-IT2F, PG-IT and PY-IT2F-based all-PSCs.

Blend	E_g(eV)	qV_{oc}^{SQ}	qV_{oc}^{rad} (eV)	ΔE_1 (eV)	ΔE_2 (eV)	ΔE_3 (eV)	E_{loss} (eV)
PM6:PG-IT2F	1.47	1.206	1.14	0.265	0.068	0.190	0.523
PM6:PG-IT	1.47	1.206	1.15	0.266	0.066	0.203	0.535
PM6:PY-IT2F	1.45	1.18	1.12	0.263	0.061	0.215	0.539

Supplementary Table 6. The parameters of exciton dissociation efficiency and charge collection efficiency in the all-PSCs.

Active layer	J_{sat} (mA/cm^2)	J_{pha} (mA/cm^2)	J_{phb} (mA/cm^2)	P_{diss} (%)	P_{coll} (%)
PM6:PY-IT2F	19.06	17.01	14.07	89.24%	73.82%
PM6:PG-IT	18.51	16.61	13.92	90.72%	76.02%
PM6:PG-IT2F	19.80	18.44	15.78	93.13%	79.70%

Supplementary Table 7. GIWAXS measurement performance parameters of the related films.

	in plane			out of plane		
Film	Location (Å^{-1})	*d*-spacing (Å)	CCL (Å)	Location (Å^{-1})	*d*-spacing (Å)	CCL (Å)
PY-IT2F	0.392	16.02	112.37	1.56	4.03	22.7
PG-IT	0.385	16.32	76.88	1.56	4.02	23.5
PG-IT2F	0.381	16.49	72.14	1.55	4.05	20.8
PM6	0.281	22.36	89.90	1.65	3.80	21.8
PM6:PY-IT2F	0.287	21.89	121.74	1.60	3.92	21.8
PM6:PG-IT	0.287	21.89	157.93	1.62	3.87	19.7
PM6:PG-IT2F	0.283	22.20	182.61	1.62	3.89	20.5

Supplementary Methods

Synthesis of PY-IT2F

The synthetic route of monomer 1 and PY-IT2F was shown in Supplementary Figures 1 and 3. Monomer 1 (100 mg, 0.05 mmol), (3,4-Difluorothiophene-2,5-diyl)bis(trimethylstannane) (0.05 mmol, 22.29 mg), $Pd(dba)_2$ (1.2 mg) and Tri(o-tolyl)phosphine (2.1 mg) are put into the pressure tube together, 5 ml toluene are added as solvent, and reacted for 48 h under argon atmosphere. The crude product PY-IT2F was obtained, and then extracted with a Soxhlet extractor. The extraction solvents were methanol, acetone, *n*-hexane, and chloroform. Finally, the pure polymer PY-IT2F was obtained. (81.6 mg, 66.72% yield, M_n =26.3 kDa).

Synthesis of PG-IT

The synthetic route of PG-IT was shown in Supplementary Figure 4. Monomer 6 (100 mg, 0.049 mmol), 2,5-bis(trimethylstannyl)thiophene (0.049 mmol, 20.08 mg), $Pd(dba)_2$ (1.2 mg) and Tri(o-tolyl)phosphine (2.1 mg) are put into the pressure tube together, 5 ml toluene are added as solvent, and reacted for 48 h under argon atmosphere. The crude product PG-IT was obtained, and then extracted with a Soxhlet extractor. The extraction solvents were methanol, acetone, *n*-hexane, and chloroform. Finally, the pure polymer PG-IT was obtained. (74.8 mg 62.3% yield). M_n=11.9 kDa.

Synthesis of PG-IT2F

The synthetic route of PG-IT2F was shown in Supplementary Figure 5. Monomer 6 (100 mg, 0.049 mmol), (3,4-Difluorothiophene-2,5-diyl)bis(trimethylstannane) (0.049 mmol, 21.84 mg), $Pd(dba)_2$ (1.2 mg) and Tri(o-tolyl)phosphine (2.1 mg) are put into the

pressure tube together, 5 ml toluene are added as solvent, and reacted for 48 h under argon atmosphere. The crude product PG-IT2F was obtained, and then extracted with a Soxhlet extractor. The extraction solvents were methanol, acetone, *n*-hexane, and chloroform. Finally, the pure polymer PG-IT2F was obtained. (78.2 mg 64.2% yield). M_n=17.1 kDa

Synthesis of Compound 4

The synthetic route of compound 4 was shown in Supplementary Figure 2. Compound 3 (1.81 g, 2.34 mmol) was mixed with K_2CO_3 (3.23 g, 23.4 mmol), KI (0.39 g, 2.34 mmol), 11-(bromomethyl) tricosane (2.93 g, 7.02 mmol), and anhydrous DMF (25 mL) were mixed under argon and stirred at 100 °C overnight. The mixture was extracted with ethyl acetate for three times. The combined organic phase was washed with water followed by brine. Then, the solution was dried over Na_2SO_4 and concentrated under reduced pressure to yield an orange oil. Compound 4 was obtained by column chromatography on silica gel using petroleum ether/dichloromethane (8/1, v/v) as the eluent to give an orange oil (1.56 g, 46% yield). ^{1}H NMR (400 MHz, $CDCl_3$) δ 6.99 (s, 2H), 4.61 (d, J = 7.7 Hz, 4H), 2.76 (d, J = 7.2 Hz, 4H), 2.25 – 1.81 (m, 4H), 1.63 – 0.37 (m, 156H). ^{13}C NMR (101 MHz, $CDCl_3$) δ 147.67, 142.42, 137.10, 135.95, 131.64, 123.55, 122.78, 119.95, 111.58, 55.10, 38.65, 37.42, 34.67, 33.76, 33.42, 31.98, 31.94, 31.92, 30.50, 29.77, 29.68, 29.67, 29.60, 29.55, 29.48, 29.41, 29.37, 29.32, 28.85, 26.59, 25.55, 23.11, 22.73, 22.71, 14.13, 14.11. HRMS (TOF) m/z calcd. For $[M]^+$ $C_{90}H_{150}N_4S_5$ 1447.0464, found 1447.0459.

Synthesis of Compound 5

The synthetic route of compound 5 was shown in Supplementary Figure 2. Compound 4 (1.56 g, 1.08 mmol) was dissolved in 20 mL trichloromethane under argon, then the fresh Vilsmeier reagent (3.8 mL $POCl_3$ in 7.5 mL DMF) was added dropwise at 0 °C. After stirring for 20 min at 0 °C, the mixture was heated to 65 °C and reacted 24 h. The reaction was quenched with saturated $NaHCO_3$ solution and allowed to stir at room temperature for 24 h. The organic layer was separated, and the aqueous phase was extracted with dichloromethane for three times. The combined organic layer was washed with water, dried over Na_2SO_4, filtered, and concentrated under reduced pressure. The crude product was purified with column chromatograph on silica gel using petroleum ether/dichloromethane (2/1, v/v) as the eluent to give an orange oil Compound 5 (1.32 g, 81% yield). ^{1}H NMR (400 MHz, $CDCl_3$) δ 10.11 (s, 2H), 4.63 (d, J = 7.8 Hz, 4H), 3.09 (d, J = 7.4 Hz, 4H), 2.06 (h, J = 6.3 Hz, 4H), 1.67 – 0.33 (m, 164H). ^{13}C NMR (101 MHz, $CDCl_3$) δ 181.71, 147.48, 146.15, 143.45, 137.82, 136.83, 132.86, 129.44, 127.35, 112.46, 55.38, 39.17, 38.89, 33.87, 33.55, 33.20, 31.94, 31.91, 31.83, 30.45, 29.66, 29.64, 29.62, 29.57, 29.52, 29.47, 29.37, 29.30, 29.29, 28.80, 26.58, 25.50, 22.99, 22.70, 22.68, 22.63, 14.10, 14.06, 14.03. HRMS (TOF) m/z calcd. For $[M]^+$ $C_{92}H_{150}N_4O_2S_5$ 1503.0362, found 1503.0357.

Synthesis of Compound 6

The synthetic route of compound 6 was shown in Supplementary Figure 2. Compound 5 (100.0 mg, 0.05 mmol), 2-(5-bromo-3-oxo-2,3-dihydro-1H-inden-1-ylidene)malononitrile (81.9 mg, 0.48 mmol), pyridine (1.0 mL), and chloroform (15.0

mL) were dissolved in a round bottom flask under nitrogen. The mixture was stirred at room temperature overnight, then the mixture was poured into methanol and filtered. The residue was purified with column chromatography on silica gel using dichloromethane/petroleum ether (1/1, v/v) as the eluent to give a dark blue solid Compound 6 (74.54 mg, 74% yield). ^{1}H NMR (300 MHz, $CDCl_3$) δ 9.14 (s, 2H), 8.54 (d, J = 8.4 Hz, 2H), 8.01 (s, 2H), 7.83 (d, J = 8.5 Hz, 2H), 4.79 (d, J = 7.8 Hz, 4H), 3.14 (d, J = 7.5 Hz, 4H), 2.11 (dq, J = 20.4, 6.6 Hz, 4H), 1.68 – 0.26 (m, 144H). ^{13}C NMR (75 MHz, $CDCl_3$) δ 186.82, 160.14, 153.25, 147.56, 145.29, 138.49, 138.32, 137.59, 135.94, 135.74, 134.32, 134.17, 130.86, 129.44, 126.69, 126.35, 120.23, 115.47, 114.95, 113.50, 68.35, 55.77, 40.03, 39.19, 34.69, 33.59, 33.33, 31.97, 31.95, 31.88, 30.62, 29.79, 29.73, 29.67, 29.59, 29.47, 29.43, 29.39, 28.86, 26.59, 25.66, 23.03, 22.73, 22.68, 14.15, 14.12, 14.09. HRMS (TOF) m/z calcd. For $[M]^+$ $C_{116}H_{156}Br_2N_8O_2S_5$ 2010.9301, found 2010.9316.

Supplementary References

1. Luo Z. et al. Precisely controlling the position of bromine on the end group enables well-regular polymer acceptors for all-polymer solar cells with efficiencies over 15%. *Adv. Mater.* **32**, 2005942 (2020).
